# Barcoding Bugs: DNA-Based Identification of the True Bugs (Insecta: Hemiptera: Heteroptera)

**DOI:** 10.1371/journal.pone.0018749

**Published:** 2011-04-15

**Authors:** Doo-Sang Park, Robert Foottit, Eric Maw, Paul D. N. Hebert

**Affiliations:** 1 Biological Resource Center, Korea Research Institute of Bioscience and Biotechnology, Daejeon, Korea; 2 Agriculture and Agri-Food Canada, Invertebrate Biodiversity – National Environmental Health Program, and Canadian National Collection of Insects, Arachnids and Nematodes, Ottawa, Ontario, Canada; 3 Biodiversity Institute of Ontario, University of Guelph, Guelph, Ontario, Canada; Natural History Museum of Denmark, Denmark

## Abstract

**Background:**

DNA barcoding, the analysis of sequence variation in the 5′ region of the mitochondrial cytochrome *c* oxidase I (COI) gene, has been shown to provide an efficient method for the identification of species in a wide range of animal taxa. In order to assess the effectiveness of barcodes in the discrimination of Heteroptera, we examined 344 species belonging to 178 genera, drawn from specimens in the Canadian National Collection of Insects.

**Methodology/Principal Findings:**

Analysis of the COI gene revealed less than 2% intra-specific divergence in 90% of the taxa examined, while minimum interspecific distances exceeded 3% in 77% of congeneric species pairs. Instances where barcodes fail to distinguish species represented clusters of morphologically similar species, except one case of barcode identity between species in different genera. Several instances of deep intraspecific divergence were detected suggesting possible cryptic species.

**Conclusions/Significance:**

Although this analysis encompasses 0.8% of the described global fauna, our results indicate that DNA barcodes will aid the identification of Heteroptera. This advance will be useful in pest management, regulatory and environmental applications and will also reveal species that require further taxonomic research.

## Introduction

The true bugs (Insecta: Hemiptera: Heteroptera) represent the largest group of hemimetabolous insects, with more than 42,000 described species in over 5800 genera and 140 families [1]. The order includes many economically important plant pests, animal disease vectors and predators employed in biological control [1], [2]. Among the Heteroptera, there are a number of taxonomically difficult groups which include pest species (for example *Lygus* species [3]). As well, immature forms are generally difficult to identify using morphology-based keys.

The 5′ end of the mitochondrial cytochrome *c* oxidase subunit I gene (COI) has been proposed as a standardized DNA “barcode” for the identification of species in the animal kingdom [4], [5]. DNA barcodes could aid in the routine identification of Heteroptera in applied settings by enabling the recognition of morphologically cryptic species, by associating immature forms with adults (pest management), and by identifying eggs (phytosanitary applications) and fragmentary remains (food quality, ecological analyses).

Only a few prior studies have employed DNA sequences for species identification in the Heteroptera. Damgaard [6] found that COI sequences (in this case, from the 3′ end of the gene) were of limited utility in the identification of a *Gerris* species group. Memon *et al.* [7] confirmed the usefulness of variation in COI sequences in circumscribing a new hemipteran species, but found broad overlap in intraspecific and interspecific distances among sequences of 373 species of Hemiptera downloaded from GenBank. However, most of the latter data derive from studies specifically directed towards elucidating relationships within taxonomically problematic groups. Thus the available data are biased towards situations in which recent speciation reduces the observed level of inter-species sequence divergence, and may underestimate the utility of DNA barcoding as an identification tool among Heteroptera in general.

Recently, Jung *et al.* [8] presented COI barcode sequences for East Asian Heteroptera, and concluded that these barcodes can contribute to species identification. However, 79 of the 139 species treated were from three families (Anthocoridae (*sensu lato*), Miridae and Pentatomidae), and 11 of 25 families were represented by a single species, limiting the degree to which their conclusions may be generalized to Heteroptera as a whole. The present study expands the survey of sequence variation in the standard COI region in Heteroptera based on the analysis of identified specimens held in the Canadian National Collection of Insects.

## Materials and Methods

### Specimens

Specimens for this study were drawn from the Canadian National Collection of Insects, Arachnids and Nematodes, Ottawa. Material collected more than 40 years ago was avoided in order to maximize the sequencing success rate. Whenever possible, more than one individual of a species was selected. An attempt was made to gain representation of all major heteropteran groups available, with more intensive coverage of certain groups. Thus, about 60% of the species are from the large family Miridae, and within this family, several speciose genera or species groups which present taxonomic difficulties were sampled more densely. A total of 1689 identified specimens were examined. Most specimens were from North America, but some were from Central America and Europe. A few specimens were preserved in 95% ethanol, but most were dried, pinned specimens collected over the past three decades (median age about 11 years). Collecting data were entered into BOLD, the Barcoding of Life Data System [9] and are available in the HCNC and HCNCS (“CNC Hemiptera”) projects (http://www.barcodinglife.org). A label was added to each specimen linking it with the corresponding record on BOLD.

### CO1 Amplification and Sequencing

A single leg was removed from dried or ethanol-fixed specimens and DNA was extracted using standard glass fibre extraction protocol [10]. PCR amplifications were done in a 12.5 µl volume including 6.25 µl of 10% trehalose, 2 µl of ultra pure water, 1.25 µl of 10 × PCR buffer (10 mM KCl, 10 mM (NH_4_)_2_SO_4_, 20 mM Tris-HCl (pH 8.8), 2 mM MgSO_4_, 0.1% Triton X-100), 0.625 µl of MgCl_2_ (50 mM), 0.125 µl of each primer (10 uM), 0.0625 µl of 10 mM dNTP, 0.06 µl of Taq polymerase (Platinum® Taq, Invitrogen, CA) and 2 µl of extracted DNA. PCR primers used in this study are listed in Table 1. PCR thermocycling was performed under the following conditions: 2 min at 95°C; 5 cycles of 40 sec at 94°C, 40 sec at 45°C, 1 min at 72°C; 35 cycles of 40 sec at 94°C, 40 sec at 51°C, 1 min at 72°C; 5 min at 72°C; held at 4°C. Five additional cycles were added when using primer cocktail C_tRWF_t1 (mix of forward primers given in Table 1). PCR checks and DNA sequencing were carried out using standard methods. For about 68% of the samples, the primers LepF2_t1-3′ with a M13F tail on its 5′ end and LepR1 amplified the target 658-bp fragment of mitochondrial CO1 gene. When these primers were not successful, the primer cocktail C-tRWF_t1 (see Table 1) enabled amplification of the standard 658-bp barcode region together with a short upstream sequence in an additional 15% of the specimens. Specimens that were still recalcitrant were then amplified with the primer combination LepF2 (or C_tRWF_t1) with MHemR and MHemF with LepR1 to generate shorter overlapping sequences that allowed the creation of a composite sequence. Contigs and alignments were made using CodonCode Aligner Ver2.0.6 (CodonCode Co.). Sequence divergences were calculated using a K2P distance model [11] and a Neighbour-joining (NJ) tree [12] was generated to provide a graphic representation of the species divergences as implemented in the ‘Sequence analysis’ module on BOLD [9]. All sequences corresponding to project HCNC have been deposited in GenBank (accession numbers HM394326 to HM394342, HM914596 to HM914598, and HQ105390 to HQ106459). Collection details, specimen photographs, sequences, trace files and GenBank accession numbers are available within the HCNC and HCNCS project files in BOLD.

**Table 1:** PCR primers used in this study.

Primer Name	Primer sequence (5′-3′)	Primer source	Sequencing primer
LepF2_t1	M13F-AATCATAARGATATYGG	Modified from [17]	M13F
LepR1	TAAACTTCTGGATGTCCAAAAAATCA	[17]	LepR1
tRWF1_t1^a^	M13F-AAACTAATARCCTTCAAAG	[18]	M13F
tRWF2_t1^a^	M13F-AAACTAATAATYTTCAAAATTA	[18]	M13F
MHemF	GCATTYCCACGAATAAATAAYATAAG	New (230–255)^b^	N/A
MHemR	GGTGGATAAACTGTTCAWCC	New (343–326)^b^	N/A

a: two primers combined in cocktail primer, C-tRWF.

b: nucleotide position of standard barcode region.

## Results

### Species identification

Barcodes were obtained for about 80% of the specimens with successful amplification from specimens up to 35 years old. The 1276 sequences represent 380 species that belong to 191 genera in 30 families (Table 2). Of these, 1090 sequences (344 species, 178 genera, 29 families; see Table 3) were more than 500 bases in length. No stop codons or frame shifts were detected in the COI sequences, suggesting that none derive from pseudogenes (NUMTs). The following analysis only considers sequences with a length greater than 500 bp (see project HCNC). Shorter sequences are available in the HCNCS project, but not discussed further. The complete NJ tree derived from project HCNC is available as Appendix S1.

**Table 2:** Taxonomic placement of taxa sampled and summary of the distribution of species by sequence divergence (K2P) from their nearest neighbor at COI barcode sequence.

	number of species						
	described (world)	barcoded		minimum distance to nearest neighbour (for sequences >500 bp only)			
Family		<500 bp	>500 bp	<0.1%	>0.1 to 1%	>1 to 3%	>3%
Acanthosomatidae	184	0	1				1
Alydidae	254	0	6		3		3
Anthocoridae	534	1	2				2
Artheneidae	20	0	1				1
Berytidae	172	0	3				3
Blissidae	435	0	1				1
Cimicidae	110	0	1				1
Coreidae	1884	0	4			2	2
Corixidae	607	2	19			4	15
Cydnidae	560	1	1				1
Cymidae	54	0	1				1
Geocoridae	274	0	6		4		2
Gerridae	751	1	2				2
Lygaeidae	968	0	8				8
Mesoveliidae	46	0	1				1
Miridae	10040	19	205	9	19	29	148
Nabidae	386	1	5				5
Nepidae	268	1	2				2
Notonectidae	400	0	5				5
Oxycarenidae	147	0	1				1
Pachygronthidae	78	1	1				1
Pentatomidae	4700	0	23				23
Pleidae	38	1	0				0
Reduviidae	6878	0	5				5
Rhopalidae	209	0	10			2	8
Rhyparochromidae	1850	4	12		2		10
Saldidae	335	0	6				6
Scutelleridae	450	0	4				4
Thyreocoridae	205	1	3				3
Tingidae	2124	3	5				5
60 other families	7386	—	—				
**Totals:**	**42347**	**36**	**344**	**9**	**28**	**37**	**270**

The number of described species follows estimates in Henry[1].

**Table 3:** Sequence divergences (K2P) at the COI barcode region for Hemiptera at varied taxonomic levels.

	Range (%)	Mean Dist (%)	SE (%)
**within species**	0–7.72	0.74 (0.8)	0.027
**among species in genus**	0–24.80	10.67 (12.6)	0.074
**among genera in family**	0–35.80	19.81 (19.9)	0.007
**among families**	12.15–36.67	23.66	0.005

The results are based on the analysis of 1090 specimens from 344 species belonging to 178 genera and 29 families. Corresponding mean values from Jung *et al.* [8] are given in parentheses.

**Figure 1:**
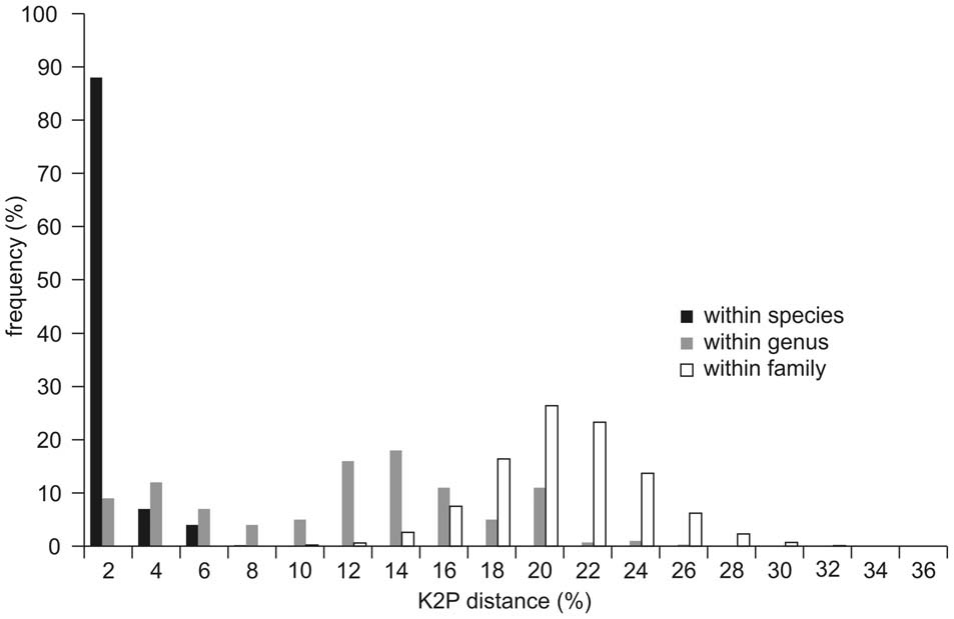
Genetic divergences (K2P distances) between COI sequences for varied taxonomic levels of Heteroptera. Frequency of pairwise divergence among specimens within species, among species within genera, and among genera within families.

Table 3 and Figure 1 summarize divergences (K2P distance) among specimens at various taxonomic levels. Intraspecific divergences averaged 0.74% (range 0–7.72%, standard deviation 1.29%), with maximum intraspecific divergence exceeding 2% in 27 of the 344 species (Table 4). Congeneric species showed an average of 10.7% divergence (range 0–24.8%) with minimum interspecific distances exceeding 3% for more than three quarters of the species pairs. The remaining species fell into two categories: species pairs that shared closely similar or identical barcodes (Table 5A), and species pairs with low sequence divergence, but forming separate clusters (Table 5B).

**Table 4:** Species with maximum intraspecific pairwise divergence (K2P) greater than 2%.

Family	species	number of specimens	intraspecific distance (%)	
			mean	maximum
Alydidae	*Alydus conspersus*	7	1.23	2.2
	*Tollius curtulus*	6	2.05	3.04
Corixidae	*Callicorixa audeni*	3	2.82	4.14
	*Sigara bicoloripennis*	6	1.43	2.73
Lygaeidae	*Kleidocerys resedae*	6	1.32	2.68
Miridae	*Adephocoris lineolatus*	11	0.87	2.65
	*Coriomeris humilis*	2	2.32	—
	*Deraeocoris bakeri*	2	2.93	—
	*Europiella artemisiae*	3	1.92	3.01
	*Labopidea lenensis*	2	4.61	—
	*Labopidea nigrisetosa*	6	0.87	2.47
	*Lygocoris pabulinus*	20	2.06	5.98
	*Macrotylus intermedius*	5	1.37	2.98
	*Orthotylus alni*	3	1.56	2.19
	*Plagiognathus morrisoni*	6	1.19	3.43
	*Plagiognathus obscurus*	16	2.44	5.57
	*Plagiognathus shoshonea*	6	1.13	2.37
	*Plagiognathus verticalis*	2	2.33	—
	*Psallus falleni*	3	5.63	7.72
	*Stenotus binotatus*	7	0.85	2.64
	*Tupiocoris rubi*	5	3.04	5.30
Pentatomidae	*Brochymena quadripustulata*	4	2.91	4.41
	*Podisus serieventris*	3	3.62	5.43
Rhopalidae	*Boisea rubrolineata*	5	1.4	3.05
	*Stictopleurus punctiventris*	11	1.09	2.34
Rhyparochromidae	*Scolopostethus thomsoni*	2	2.07	--
Scutelleridae	*Homaemus aeneifrons*	8	1.62	3.35

**Table 5:** Groups of nominal species poorly discriminated by COI barcodes.

Family	species	number of specimens	max intrasp. distance (%)	interspecific distance (%)	
				Range	mean
**A.**					
Alydidae	*Alydus conspersus/calcaratus/eurinus*	7/3/7	2.2	0.35–2.57	1.56
Coreidae	*Coriomeris humilis/insularis*	2/2	2.32	1.23–1.48	1.34
Geocoridae	*Geocoris howard/limbatus*	2/2	1.39	0.16–1.39	0.74
Miridae	*Europiella artemisiae/decolor*	3/1	3.01	1.20–3.14	1.69
	*Henrylygus nubilus/ultranubilus*	4/3	0.31	0–0.33	0.05
	*Labopidea nigrosetosa/pallida/simplex*	6/2/3	2.47	0.20–2.98	1.19
	*Lygocoris communis/inconspicuus/tinctus*	4/2/6	0.46	0.61–2.20	1.66
	*Lygus humilis/striatus*	2/2	0.46	0–0.46	0.23
	*Orthotylus affinis/alni/katmai/pacificus*	3/3/3/4	2.19	0–2.51	1.06
	*Phytocoris crawfordi/driesbachi*	3/4	1.71	1.24–1.89	1.60
	*Plagiognathus brunneus*/*obscurus* [part]*/shoshonea*	3/12/6	2.45	0–2.06	0.75
	*Plagiognathus emarginatae/fuscipes/morrisoni*	2/3/5	0.33	0–0.67	0.29
	*Rhinocapsus vanduzeei/Plagiognathus emarginatae* group^1^	3/10^1^	0.36	0–0.81	0.29
Nabidae	*Nabicula subcoleoptrata/vanduzeei*	3/2	0.48	0.15–0.32	0.30
Rhyparochromidae	*Ligyrocoris diffuses/sylvestris*	4/5	1.08	0.31–1.12	0.91
**B.**					
Corixidae	*Cenocorixa bifida/dakotensis/expleta/utahensis*	3/4/2/2	1.27	1.24–2.58	1.97
Geocoridae	*Geocoris discopterus/pallens/howardi+limbatus* ^2^	2/4/4^2^	1.39	0.77–2.82	1.79
Miridae	*Irbisia nigipes/shulli*	1/4	0	1.05–1.19	1.12
	*Labopidea ampla/nigridia*	3/2	0.17	1.95–2.68	2.21
	*Lygus elisus/hesperus/humeralis/plagiatus/rubroclarus/striatus/unctuosus*		0.46	0.31–2.22	1.37
	*Oligotylus centralis/pluto*	2/3	0.33	1.86–2.02	1.91
	*Pilophorus americanus/piceicola*	3/1	0	1.71–1.71	1.71
	*Pinalitus approximates/rostratus*	4/8	0.19	0.92–1.63	1.43
	*Plagiognathus modestus/punctatipes*	1/3	0.16	1.62–7.76	1.70
	*Slaterocoris breviatus/stygicus*	1/2	0	0.87–0.95	0.91
Rhopalidae	*Arhyssus lateralis/rubrovenosus*	2/4	1.06	1.90–2.53	2.24

**A**: species not distinguished by barcode (interspecific distances less than maximum intraspecific distances). **B**: species weakly separated by barcode (minimum interspecific distance less than 2%, but mean interspecific distances exceed maximum intraspecific distances).

1 *Plagiognathus emarginatae*, *P. fuscipes* and *P. morrisoni* specimens pooled.

2 *Geocoris howardi* and *G. limbatus* specimens pooled.

### Sequence Divergence Patterns

There was one instance of barcode sharing by members of different genera: *Rhinocapsus vanduzeei* (2 of 3 specimens) shared sequences with 2 of 5 specimens of *Plagiognathus morrisoni*, and maximum distance between specimens of *R. vanduzeei* and members of the *P. fuscipes* species group (*P. emarginatae*, *fuscipes* and *morrisoni*) was 0.806%. If *R. vanduzeei* is excluded, the minimum distance between members of different genera within a family is 5.05% (mean 19.8%, maximum 35.8%). Among the 228 species with congeneric species in the current analysis, the nearest neighbour of 193 (85%) was a congener.

**Figure 2:**
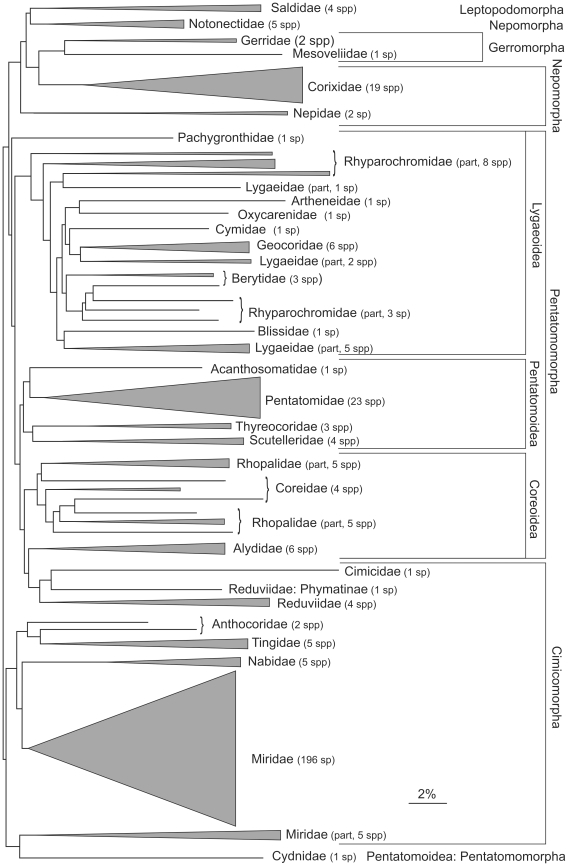
Simplified representation of affinities among families and higher taxa as shown in a neighbor-joining tree of COI divergences shown in Appendix S1.

As indicated by the NJ tree (Fig. 2 and Appendix S1), members of a particular family normally formed a coherent cluster. The principal exceptions involved members of the superfamily Lygaeoidea, the constituent families of which were, until recently, usually included in a more broadly defined family Lygaeidae. Similarly, members of the Coreoid families Coreidae and Rhopalidae were not separated into cohesive clusters by the barcode results. On the other hand, the largest (and best sampled) family, Miridae, formed a relatively cohesive group, with only two genera, *Tupiocoris* and *Usingerella*, somewhat remote from the rest of the family. The sequence distance between specimens in different families was always great than 12% (mean 23.67%, range 12.2–36.7%).

## Discussion

This study complements the strong representation of the Anthocoridae (*sensu lato*) and Pentaomidae in the taxonomic coverage of the work of Jung *et al.* [8] by providing greater representation of the aquatic Heteroptera and Lygaeoid families, and a more extensive treatment of the important family Miridae. The broad patterns of intraspecific versus interspecific divergence obtained here confirm the values reported by Jung *et al.* [8] (their reported mean values are included in Table 3). In general, COI barcodes for each species formed a distinct cluster separated from its nearest neighbour, but there were exceptions. Some of these cases involved unusually large intraspecific distances (Table 4) while others involved cases of little or no separation between species (Table 5). Where barcodes failed to distinguish species, the taxa involved were ordinarily morphologically similar and closely related. However, there was one exception; *Rhinocapsus vanduzeei* shared the same COI sequence as some members of the *Plagiognathus fuscipes* species group. All species involved in the *Rhinocapsus*/*Plagionathus* cluster were represented by more than one individual, making it unlikely that cross-contamination or misplacement of specimens during processing had occurred.

**Figure 3:**
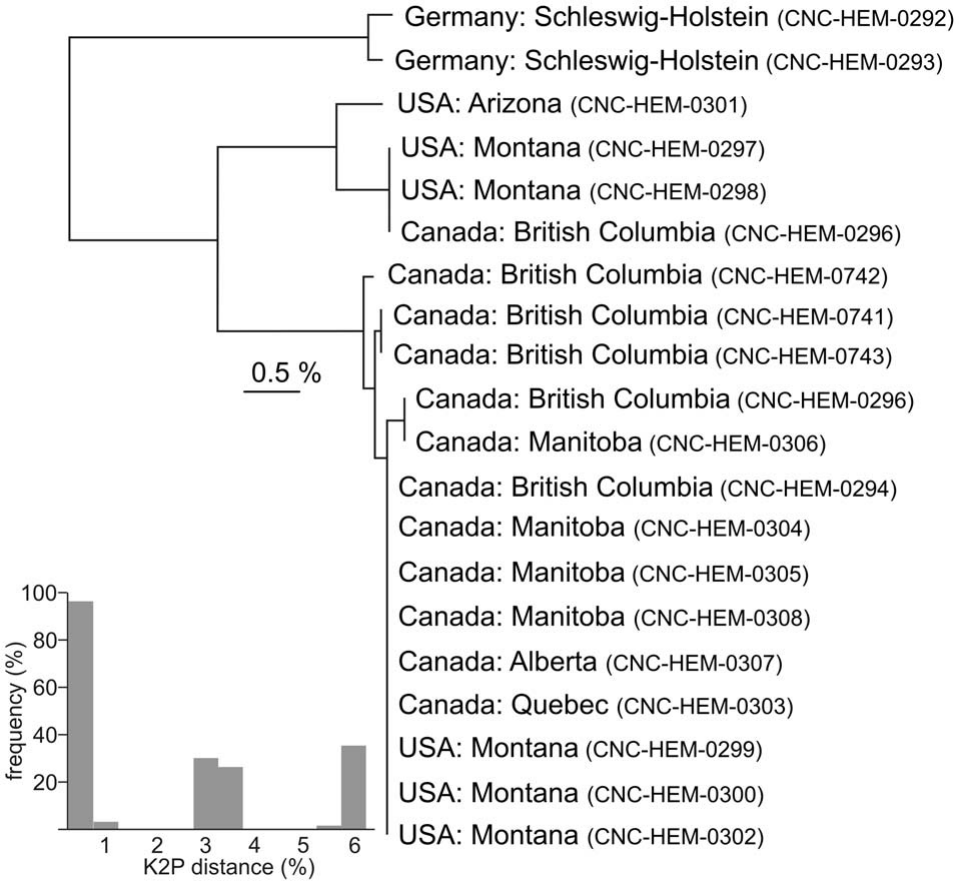
Neighbor-joining tree(K2P) showing sequence divergences at COI for specimens of *Lygocoris pabulinu*s from varied geographic localities and a plot of pairwise inter-specimen distances. Specimen data are available on BOLD through the specimen identifiers.

Cases of deep intraspecific divergence (Table 4) can reflect misidentifications, cryptic taxa, ancestral polymorphisms, or introgression. However, past studies have shown that many of these cases involve cryptic species and there was evidence for their presence in several of the present cases. For example, specimens of *Homaemus aeneifrons* fell into two groups, one consisting of specimens from eastern Canada, the other from western Canada. The western subspecies, *H. aeneifrons extensus*, possesses distinct male genitalic characters [13], and this case of deep sequence difference supports the treatment of the subspecies as distinct sibling species. *Lygocoris pabulinus* is a widespread Holarctic species with no accepted subspecies. However, we detected marked sequence divergence (maximum  =  5.98%) among the 20 specimens, and this variation fell into three groups separated by more than 2.98% versus a maximum within-group divergence of 0.96% (Fig. 3). One of these groups included specimens from Germany, the second was collected from across North America (British Columbia to Ontario), and the third from western North America (British Columbia to Arizona), suggesting unrecognized species may be present. *Tupiocoris rubi* illustrates an example of deep barcode differences associated with a biological difference. Members of this species fell into two groups: two specimens with identical barcodes were collected on blackberry, but they were 5% divergent from three specimens found on currant, suggesting that two host-specific taxa are involved (differing morphologically from another species on currant, *T. ribesi*, not included in the current study). Specimens of *Psallus falleni* (all from Vancouver Island, British Columbia, Canada) also fell into two very distinct haplotype groups with 7.6% divergence, suggesting that this species should also be examined further. Among the Korean species treated by Jung *et al.* [8], there was one example of unusually large divergence within a putative species (the Anthocorid, *Scolopocelis albodecussata*, with one individual differing by at least 12% from the remaining specimens), attributed to the possible existence of a cryptic species.

**Figure 4:**
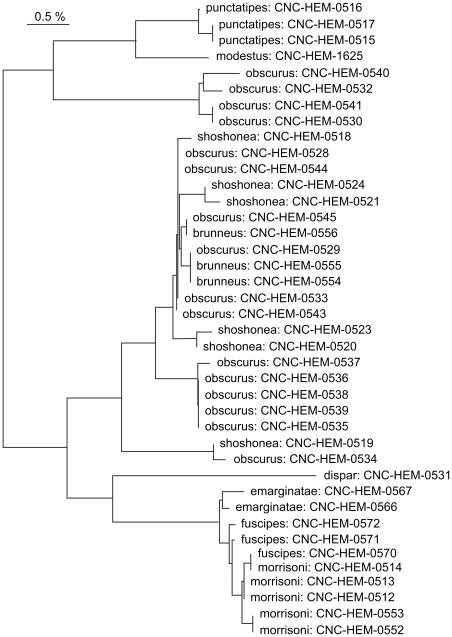
Neighbor-joining tree for specimens of selected *Plagiognathus* species (K2P). Specimen data are available on BOLD through the specimen identifiers.

In contrast to cases of deep intra-specific divergence, members of certain species complexes showed sequence sharing. Significantly, some of the species in these complexes showed high variation, a result which might reflect introgression or misidentification. As a consequence, these groups (e.g. *Plagiognathus obscurus* group, *Labopidea nigrosetosa* group, *Orthotylus alni* group) appear both as cases of high intraspecific variation (Table 4) and as cases of failed taxon discrimination (Table 5a). *Plagiognathus obscurus* and species closely related to it showed patterns of sequence variation that conflicted with current taxonomic assignments (Fig. 4) although its taxonomy was recently revised [14] and the specimens in our study were identified to reflect this treatment. However, specimens of *P. obscurus* fell into two groups separated by a minimum distance of 4.37%, contrasting with a maximum of 2.45% within-group divergence, a result suggesting cryptic species. The more diverse of these two groups of *P. obscurus* samples is intermixed with samples of *Plagiognathus brunneus* and *Plagiognathus shoshonea*. Because these species have an aggregate maximum divergence of almost 2.5%, they may represent a case of shared ancestral polymorphisms, or of species with past histories of divergence that are now introgressing. Two of these species, *P. obscurus* and *P. brunneus,* are morphologically very similar, so that misidentification is certainly a possible explanation. However, *P. shoshonea* is fairly easily recognized by its larger size, shape of male genitalia, host association, and color pattern. Misidentification of this species is therefore unlikely to have contributed to the observed patterns. Other species in this genus, such as *Plagiognathus emarginatae*, *Plagiognathus fuscipes* and *Plagiognathus morrisoni,* are morphologically similar to each other and indistinguishable by barcodes, probably reflecting recent speciation. Among the Korean species treated by Jung et al. [8], three of six nominal *Apolygus* species (Miridae) formed a single complex neighbour-joining cluster.

Close similarity of DNA barcodes among members of different genera (*Plagiognathus* and Rhinocapsus) is unusual but not unreported in other insect groups. Hausmann *et al.* [15] found COI sequence sharing among several species of the Geometrid (Lepidoptera) genera *Elophus* and *Sciadia*. Specimens of the aphids *Aulacorthum dorsatum* and *Ericaphis wakibae* (Foottit et al. [16]) have identical barcode sequences. In both cases, the authors suggest that the generic definitions require re-evaluation. This is a possible explanation for the situation encountered here, despite the obvious morphological differences currently used to distinguish the genera. However, other mechanisms are also possible, including character convergence, introgression, and lateral transfer mediated by microbial symbionts or pathogens.

Patterns of barcode similarity (Fig. 2 and Appendix S1) show a surprising congruence with current hypotheses of higher-level taxonomic relationships. In genera with more than one species in our data set, the nearest neighbour for 86% of these species was a congener. For families represented by more than one species, the nearest neighbour for all but eight species was in the same family. Because of these patterns, COI barcodes can be an indicator of generic or family-level affinity of unknown taxa, especially useful when fragmentary remains or immature forms are involved. In our experience, COI divergences of less than 5% generally provide a good indication of generic identity. When compared against the remainder of the data set using this 5% threshold, 91 species (26%) were correctly identified to genus, 1 was misidentified, and the other 252 species remained unplaced (116 of these due to the lack of congeners in the data set). Similarly, use of a 10% divergence threshold placed 48% of species in the correct family, and none were misplaced.

This study contributes to the assembly of a DNA barcode library for the Heteroptera. Although less than 1% of the world fauna has been analyzed, the present data indicate that COI barcoding provides a useful identification tool for this group. Subsequent expansion of the database to cover all important groups of Heteroptera will make it possible to reliably and routinely identify species of environmental and economic importance.

## Supporting Information

Appendix S1 Neighbour-joining tree (K2P distances) for 1090 COI sequences greater than 500 bases in length from 340 species of Heteroptera. Collection data, sequences, and trace files are available on BOLD in the HCNC project at http://www.boldsystems.
(PDF)
