# Supplementary material for: Barcoding Bugs: DNA-Based Identification of the True Bugs (Insecta: Hemiptera: Heteroptera)
Source: PLoS One. 2011 Apr 15;6(4):e18749. doi: 10.1371/journal.pone.0018749 (PMC3078146; doi:10.1371/journal.pone.0018749)
Supplement: Appendix S1 — Neighbour-joining tree (K2P distances) for 1090 COI sequences greater than 500 bases in length from 340 species of Heteroptera. Collection data, sequences, and trace files are available on BOLD in the HCNC project at http://www.boldsystems. (PDF) [file pone.0018749.s001.pdf]

BOLD TaxonID Tree

Project	: Hemiptera of Canadian National Collections [HCNC]
Date	: 5-February-2010
Data Type	: Nucleotide
Distance Model	: Kimura 2 Parameter
Codon Positions	: 1st, 2nd, 3rd
Labels	: SampleID,
Colorization	:

Sequence Count	: 1111
Species count	: 352
Genus count	: 178
Family count	: 29
Unidentified	: 0

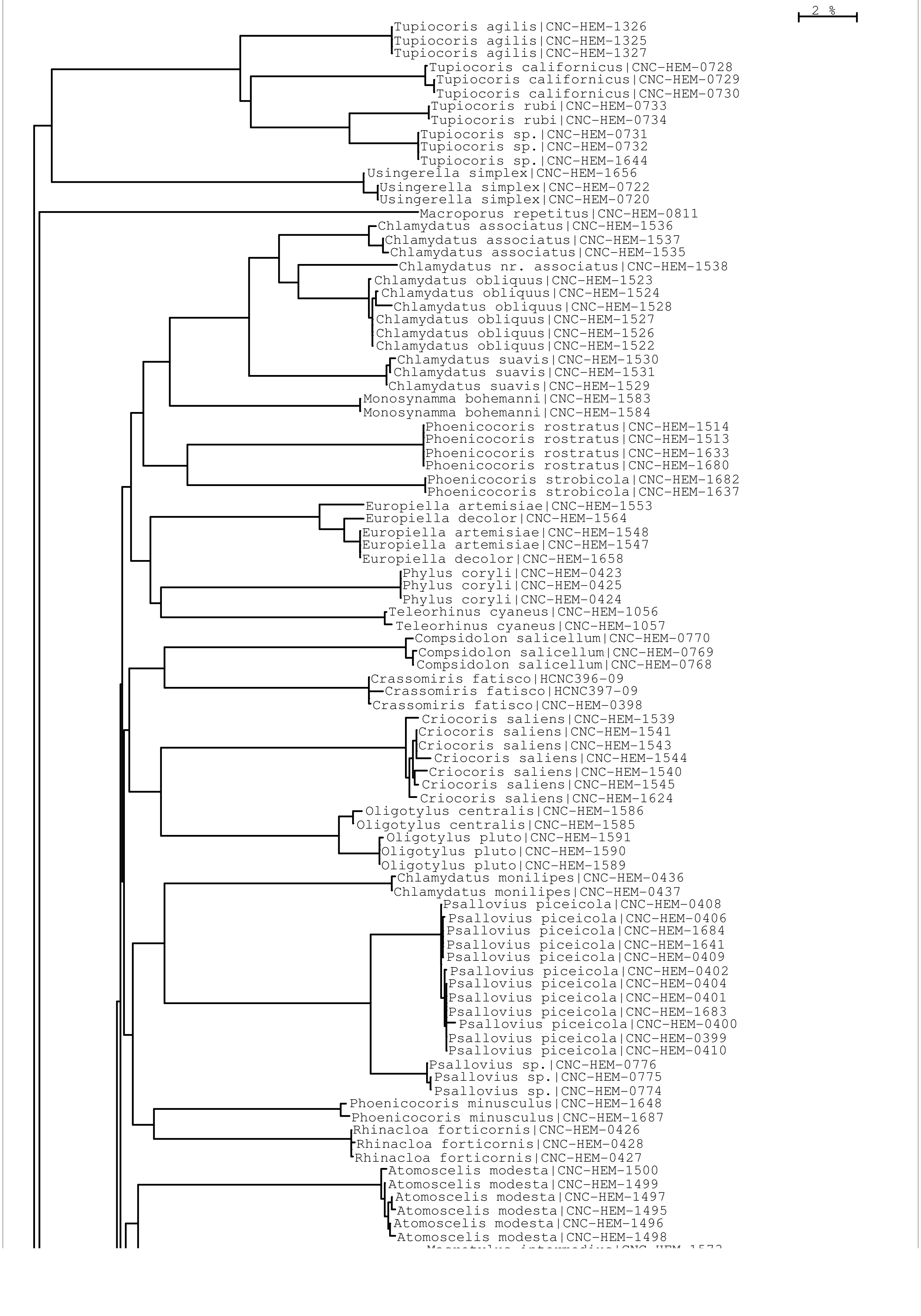
2 %
Tupiocoris agilis|CNC-HEM-1326
Tupiocoris agilis|CNC-HEM-1325
Tupiocoris agilis|CNC-HEM-1327
Tupiocoris californicus|CNC-HEM-0728
Tupiocoris californicus|CNC-HEM-0729
Tupiocoris californicus|CNC-HEM-0730
Tupiocoris rubi|CNC-HEM-0733
Tupiocoris rubi|CNC-HEM-0734
Tupiocoris sp.|CNC-HEM-0731
Tupiocoris sp.|CNC-HEM-0732
Tupiocoris sp.|CNC-HEM-1644
Usingerella simplex|CNC-HEM-1656
Usingerella simplex|CNC-HEM-0722
Usingerella simplex|CNC-HEM-0720
Macroporus repetitus|CNC-HEM-0811
Chlamydatus associatus|CNC-HEM-1536
Chlamydatus associatus|CNC-HEM-1537
Chlamydatus associatus|CNC-HEM-1535
Chlamydatus nr. associatus|CNC-HEM-1538
Chlamydatus obliquus|CNC-HEM-1523
Chlamydatus obliquus|CNC-HEM-1524
Chlamydatus obliquus|CNC-HEM-1528
Chlamydatus obliquus|CNC-HEM-1527
Chlamydatus obliquus|CNC-HEM-1526
Chlamydatus obliquus|CNC-HEM-1522
Chlamydatus suavis|CNC-HEM-1530
Chlamydatus suavis|CNC-HEM-1531
Chlamydatus suavis|CNC-HEM-1529
Monosynamma bohemanni|CNC-HEM-1583
Monosynamma bohemanni|CNC-HEM-1584
Phoenicocoris rostratus|CNC-HEM-1514
Phoenicocoris rostratus|CNC-HEM-1513
Phoenicocoris rostratus|CNC-HEM-1633
Phoenicocoris rostratus|CNC-HEM-1680
Phoenicocoris strobicola|CNC-HEM-1682
Phoenicocoris strobicola|CNC-HEM-1637
Europiella artemisiae|CNC-HEM-1553
Europiella decolor|CNC-HEM-1564
Europiella artemisiae|CNC-HEM-1548
Europiella artemisiae|CNC-HEM-1547
Europiella decolor|CNC-HEM-1658
Phylus coryli|CNC-HEM-0423
Phylus coryli|CNC-HEM-0425
Phylus coryli|CNC-HEM-0424
Teleorhinus cyaneus|CNC-HEM-1056
Teleorhinus cyaneus|CNC-HEM-1057
Compsidolon salicellum|CNC-HEM-0770
Compsidolon salicellum|CNC-HEM-0769
Compsidolon salicellum|CNC-HEM-0768
Crassomiris fatisco|HCNC396-09
Crassomiris fatisco|HCNC397-09
Crassomiris fatisco|CNC-HEM-0398
Criocoris saliens|CNC-HEM-1539
Criocoris saliens|CNC-HEM-1541
Criocoris saliens|CNC-HEM-1543
Criocoris saliens|CNC-HEM-1544
Criocoris saliens|CNC-HEM-1540
Criocoris saliens|CNC-HEM-1545
Criocoris saliens|CNC-HEM-1624
Oligotylus centralis|CNC-HEM-1586
Oligotylus centralis|CNC-HEM-1585
Oligotylus pluto|CNC-HEM-1591
Oligotylus pluto|CNC-HEM-1590
Oligotylus pluto|CNC-HEM-1589
Chlamydatus monilipes|CNC-HEM-0436
Chlamydatus monilipes|CNC-HEM-0437
Psallovius piceicola|CNC-HEM-0408
Psallovius piceicola|CNC-HEM-0406
Psallovius piceicola|CNC-HEM-1684
Psallovius piceicola|CNC-HEM-1641
Psallovius piceicola|CNC-HEM-0409
Psallovius piceicola|CNC-HEM-0402
Psallovius piceicola|CNC-HEM-0404
Psallovius piceicola|CNC-HEM-0401
Psallovius piceicola|CNC-HEM-1683
Psallovius piceicola|CNC-HEM-0400
Psallovius piceicola|CNC-HEM-0399
Psallovius piceicola|CNC-HEM-0410
Psallovius sp.|CNC-HEM-0776
Psallovius sp.|CNC-HEM-0775
Psallovius sp.|CNC-HEM-0774
Phoenicocoris minusculus|CNC-HEM-1648
Phoenicocoris minusculus|CNC-HEM-1687
Rhinacloa forticornis|CNC-HEM-0426
Rhinacloa forticornis|CNC-HEM-0428
Rhinacloa forticornis|CNC-HEM-0427
Atomoscelis modesta|CNC-HEM-1500
Atomoscelis modesta|CNC-HEM-1499
Atomoscelis modesta|CNC-HEM-1497
Atomoscelis modesta|CNC-HEM-1495
Atomoscelis modesta|CNC-HEM-1496
Atomoscelis modesta|CNC-HEM-1498

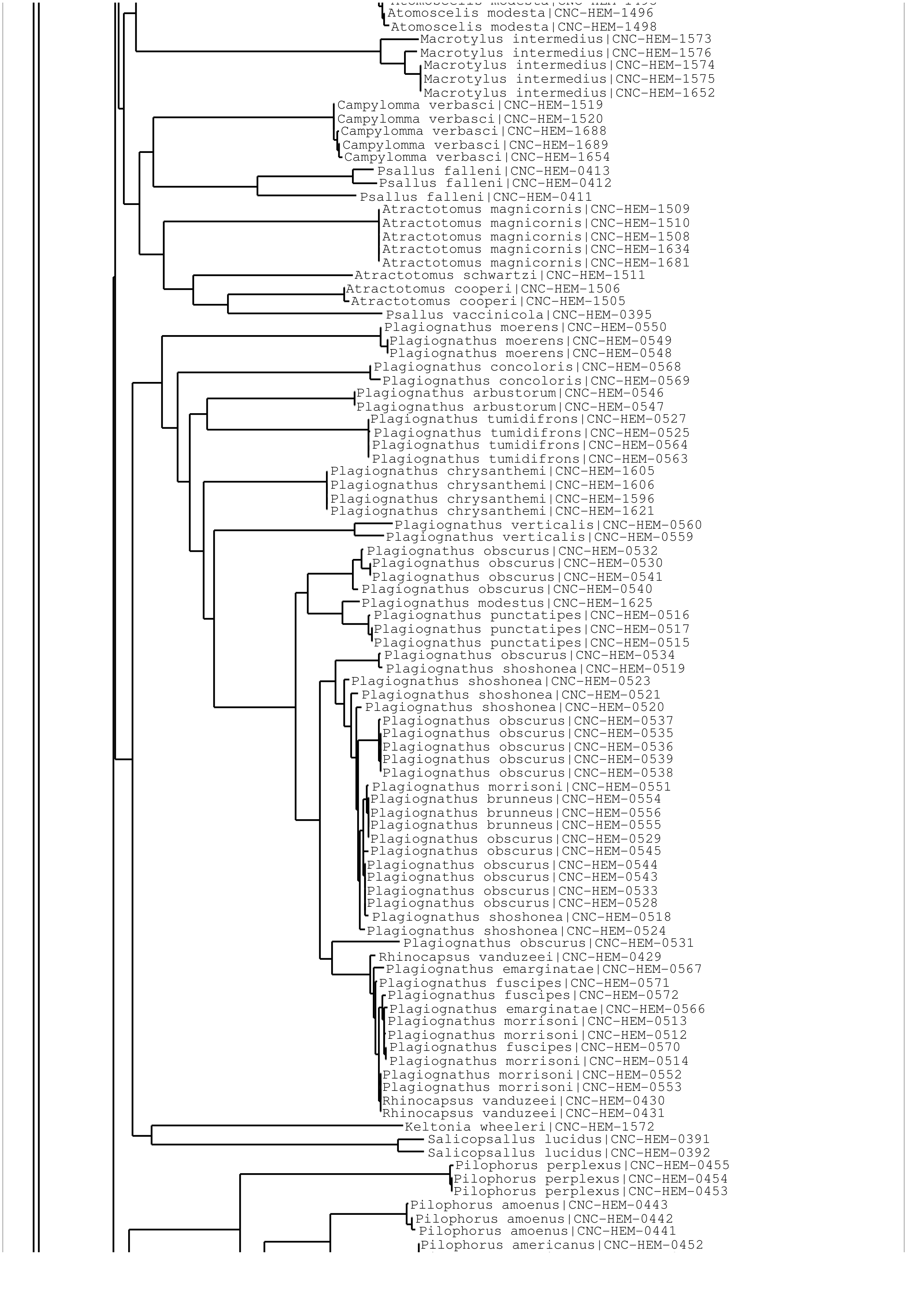

Atomoscelis modesta|CNC-HEM-1496
Atomoscelis modesta|CNC-HEM-1498
Macrotylus intermedius|CNC-HEM-1573
Macrotylus intermedius|CNC-HEM-1576
Macrotylus intermedius|CNC-HEM-1574
Macrotylus intermedius|CNC-HEM-1575
Macrotylus intermedius|CNC-HEM-1652
Campylomma verbasci|CNC-HEM-1519
Campylomma verbasci|CNC-HEM-1520
Campylomma verbasci|CNC-HEM-1688
Campylomma verbasci|CNC-HEM-1689
Campylomma verbasci|CNC-HEM-1654
Psallus falleni|CNC-HEM-0413
Psallus falleni|CNC-HEM-0412
Psallus falleni|CNC-HEM-0411
Atractotomus magnicornis|CNC-HEM-1509
Atractotomus magnicornis|CNC-HEM-1510
Atractotomus magnicornis|CNC-HEM-1508
Atractotomus magnicornis|CNC-HEM-1634
Atractotomus magnicornis|CNC-HEM-1681
Atractotomus schwartzi|CNC-HEM-1511
Atractotomus cooperi|CNC-HEM-1506
Atractotomus cooperi|CNC-HEM-1505
Psallus vaccinicola|CNC-HEM-0395
Plagiognathus moerens|CNC-HEM-0550
Plagiognathus moerens|CNC-HEM-0549
Plagiognathus moerens|CNC-HEM-0548
Plagiognathus concoloris|CNC-HEM-0568
Plagiognathus concoloris|CNC-HEM-0569
Plagiognathus arbustorum|CNC-HEM-0546
Plagiognathus arbustorum|CNC-HEM-0547
Plagiognathus tumidifrons|CNC-HEM-0527
Plagiognathus tumidifrons|CNC-HEM-0525
Plagiognathus tumidifrons|CNC-HEM-0564
Plagiognathus tumidifrons|CNC-HEM-0563
Plagiognathus chrysanthemi|CNC-HEM-1605
Plagiognathus chrysanthemi|CNC-HEM-1606
Plagiognathus chrysanthemi|CNC-HEM-1596
Plagiognathus chrysanthemi|CNC-HEM-1621
Plagiognathus verticalis|CNC-HEM-0560
Plagiognathus verticalis|CNC-HEM-0559
Plagiognathus obscurus|CNC-HEM-0532
Plagiognathus obscurus|CNC-HEM-0530
Plagiognathus obscurus|CNC-HEM-0541
Plagiognathus obscurus|CNC-HEM-0540
Plagiognathus modestus|CNC-HEM-1625
Plagiognathus punctatipes|CNC-HEM-0516
Plagiognathus punctatipes|CNC-HEM-0517
Plagiognathus punctatipes|CNC-HEM-0515
Plagiognathus obscurus|CNC-HEM-0534
Plagiognathus shoshonea|CNC-HEM-0519
Plagiognathus shoshonea|CNC-HEM-0523
Plagiognathus shoshonea|CNC-HEM-0521
Plagiognathus shoshonea|CNC-HEM-0520
Plagiognathus obscurus|CNC-HEM-0537
Plagiognathus obscurus|CNC-HEM-0535
Plagiognathus obscurus|CNC-HEM-0536
Plagiognathus obscurus|CNC-HEM-0539
Plagiognathus obscurus|CNC-HEM-0538
Plagiognathus morrisoni|CNC-HEM-0551
Plagiognathus brunneus|CNC-HEM-0554
Plagiognathus brunneus|CNC-HEM-0556
Plagiognathus brunneus|CNC-HEM-0555
Plagiognathus obscurus|CNC-HEM-0529
Plagiognathus obscurus|CNC-HEM-0545
Plagiognathus obscurus|CNC-HEM-0544
Plagiognathus obscurus|CNC-HEM-0543
Plagiognathus obscurus|CNC-HEM-0533
Plagiognathus obscurus|CNC-HEM-0528
Plagiognathus shoshonea|CNC-HEM-0518
Plagiognathus shoshonea|CNC-HEM-0524
Plagiognathus obscurus|CNC-HEM-0531
Rhinocapsus vanduzeei|CNC-HEM-0429
Plagiognathus emarginatae|CNC-HEM-0567
Plagiognathus fuscipes|CNC-HEM-0571
Plagiognathus fuscipes|CNC-HEM-0572
Plagiognathus emarginatae|CNC-HEM-0566
Plagiognathus morrisoni|CNC-HEM-0513
Plagiognathus morrisoni|CNC-HEM-0512
Plagiognathus fuscipes|CNC-HEM-0570
Plagiognathus morrisoni|CNC-HEM-0514
Plagiognathus morrisoni|CNC-HEM-0552
Plagiognathus morrisoni|CNC-HEM-0553
Rhinocapsus vanduzeei|CNC-HEM-0430
Rhinocapsus vanduzeei|CNC-HEM-0431
Keltonia wheeleri|CNC-HEM-1572
Salicopsallus lucidus|CNC-HEM-0391
Salicopsallus lucidus|CNC-HEM-0392
Pilophorus perplexus|CNC-HEM-0455
Pilophorus perplexus|CNC-HEM-0454
Pilophorus perplexus|CNC-HEM-0453
Pilophorus amoenus|CNC-HEM-0443
Pilophorus amoenus|CNC-HEM-0442
Pilophorus amoenus|CNC-HEM-0441
Pilophorus americanus|CNC-HEM-0452

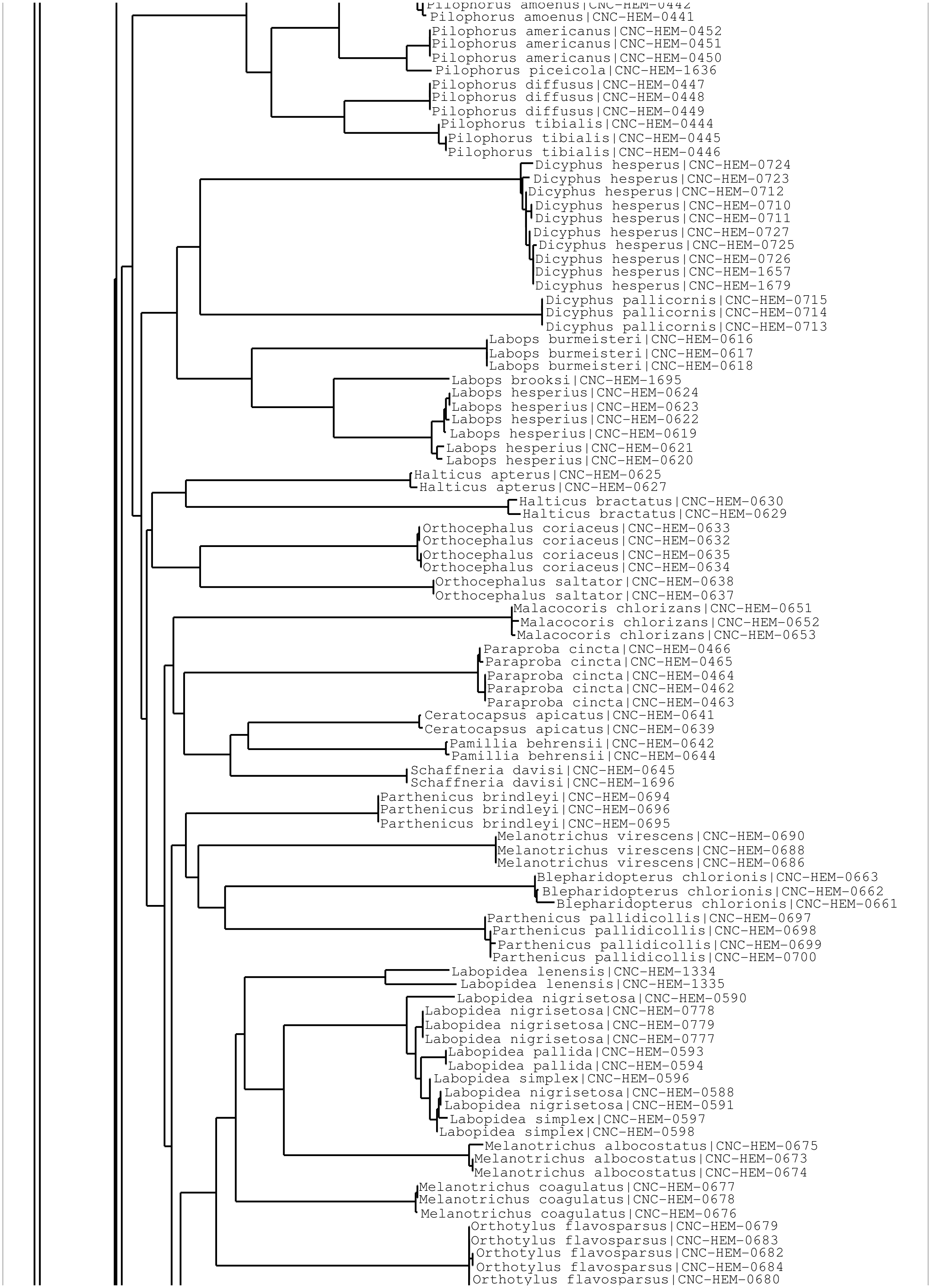
Pilophorus amoenus|CNC-HEM-0442
Pilophorus amoenus|CNC-HEM-0441
Pilophorus americanus|CNC-HEM-0452
Pilophorus americanus|CNC-HEM-0451
Pilophorus americanus|CNC-HEM-0450
Pilophorus piceicola|CNC-HEM-1636
Pilophorus diffusus|CNC-HEM-0447
Pilophorus diffusus|CNC-HEM-0448
Pilophorus diffusus|CNC-HEM-0449
Pilophorus tibialis|CNC-HEM-0444
Pilophorus tibialis|CNC-HEM-0445
Pilophorus tibialis|CNC-HEM-0446
Dicyphus hesperus|CNC-HEM-0724
Dicyphus hesperus|CNC-HEM-0723
Dicyphus hesperus|CNC-HEM-0712
Dicyphus hesperus|CNC-HEM-0710
Dicyphus hesperus|CNC-HEM-0711
Dicyphus hesperus|CNC-HEM-0727
Dicyphus hesperus|CNC-HEM-0725
Dicyphus hesperus|CNC-HEM-0726
Dicyphus hesperus|CNC-HEM-1657
Dicyphus hesperus|CNC-HEM-1679
Dicyphus pallicornis|CNC-HEM-0715
Dicyphus pallicornis|CNC-HEM-0714
Dicyphus pallicornis|CNC-HEM-0713
Labops burmeisteri|CNC-HEM-0616
Labops burmeisteri|CNC-HEM-0617
Labops burmeisteri|CNC-HEM-0618
Labops brooksi|CNC-HEM-1695
Labops hesperius|CNC-HEM-0624
Labops hesperius|CNC-HEM-0623
Labops hesperius|CNC-HEM-0622
Labops hesperius|CNC-HEM-0619
Labops hesperius|CNC-HEM-0621
Labops hesperius|CNC-HEM-0620
Halticus apterus|CNC-HEM-0625
Halticus apterus|CNC-HEM-0627
Halticus bractatus|CNC-HEM-0630
Halticus bractatus|CNC-HEM-0629
Orthocephalus coriaceus|CNC-HEM-0633
Orthocephalus coriaceus|CNC-HEM-0632
Orthocephalus coriaceus|CNC-HEM-0635
Orthocephalus coriaceus|CNC-HEM-0634
Orthocephalus saltator|CNC-HEM-0638
Orthocephalus saltator|CNC-HEM-0637
Malacocoris chlorizans|CNC-HEM-0651
Malacocoris chlorizans|CNC-HEM-0652
Malacocoris chlorizans|CNC-HEM-0653
Paraproba cincta|CNC-HEM-0466
Paraproba cincta|CNC-HEM-0465
Paraproba cincta|CNC-HEM-0464
Paraproba cincta|CNC-HEM-0462
Paraproba cincta|CNC-HEM-0463
Ceratocapsus apicatus|CNC-HEM-0641
Ceratocapsus apicatus|CNC-HEM-0639
Pamillia behrensii|CNC-HEM-0642
Pamillia behrensii|CNC-HEM-0644
Schaffneria davisi|CNC-HEM-0645
Schaffneria davisi|CNC-HEM-1696
Parthenicus brindleyi|CNC-HEM-0694
Parthenicus brindleyi|CNC-HEM-0696
Parthenicus brindleyi|CNC-HEM-0695
Melanotrichus virescens|CNC-HEM-0690
Melanotrichus virescens|CNC-HEM-0688
Melanotrichus virescens|CNC-HEM-0686
Blepharidopterus chlorionis|CNC-HEM-0663
Blepharidopterus chlorionis|CNC-HEM-0662
Blepharidopterus chlorionis|CNC-HEM-0661
Parthenicus pallidicollis|CNC-HEM-0697
Parthenicus pallidicollis|CNC-HEM-0698
Parthenicus pallidicollis|CNC-HEM-0699
Parthenicus pallidicollis|CNC-HEM-0700
Labopidea lenensis|CNC-HEM-1334
Labopidea lenensis|CNC-HEM-1335
Labopidea nigrisetosa|CNC-HEM-0590
Labopidea nigrisetosa|CNC-HEM-0778
Labopidea nigrisetosa|CNC-HEM-0779
Labopidea nigrisetosa|CNC-HEM-0777
Labopidea pallida|CNC-HEM-0593
Labopidea pallida|CNC-HEM-0594
Labopidea simplex|CNC-HEM-0596
Labopidea nigrisetosa|CNC-HEM-0588
Labopidea nigrisetosa|CNC-HEM-0591
Labopidea simplex|CNC-HEM-0597
Labopidea simplex|CNC-HEM-0598
Melanotrichus albocostatus|CNC-HEM-0675
Melanotrichus albocostatus|CNC-HEM-0673
Melanotrichus albocostatus|CNC-HEM-0674
Melanotrichus coagulatus|CNC-HEM-0677
Melanotrichus coagulatus|CNC-HEM-0678
Melanotrichus coagulatus|CNC-HEM-0676
Orthotylus flavosparsus|CNC-HEM-0679
Orthotylus flavosparsus|CNC-HEM-0683
Orthotylus flavosparsus|CNC-HEM-0682
Orthotylus flavosparsus|CNC-HEM-0684
Orthotylus flavosparsus|CNC-HEM-0680

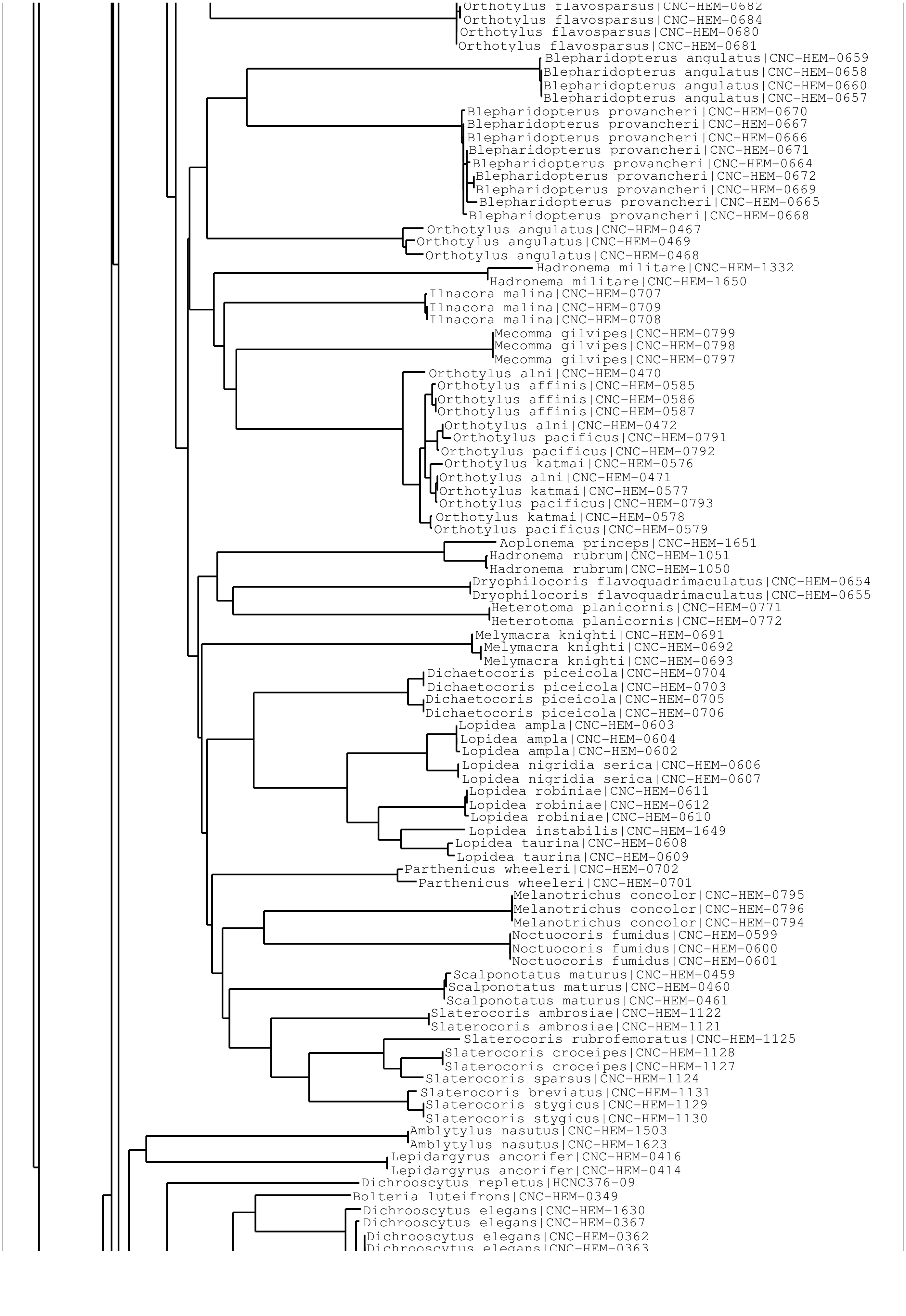
Orthotylus flavosparsus|CNC-HEM-0682
Orthotylus flavosparsus|CNC-HEM-0684
Orthotylus flavosparsus|CNC-HEM-0680
Orthotylus flavosparsus|CNC-HEM-0681
Blepharidopterus angulatus|CNC-HEM-0659
Blepharidopterus angulatus|CNC-HEM-0658
Blepharidopterus angulatus|CNC-HEM-0660
Blepharidopterus angulatus|CNC-HEM-0657
Blepharidopterus provancheri|CNC-HEM-0670
Blepharidopterus provancheri|CNC-HEM-0667
Blepharidopterus provancheri|CNC-HEM-0666
Blepharidopterus provancheri|CNC-HEM-0671
Blepharidopterus provancheri|CNC-HEM-0664
Blepharidopterus provancheri|CNC-HEM-0672
Blepharidopterus provancheri|CNC-HEM-0669
Blepharidopterus provancheri|CNC-HEM-0665
Blepharidopterus provancheri|CNC-HEM-0668
Orthotylus angulatus|CNC-HEM-0467
Orthotylus angulatus|CNC-HEM-0469
Orthotylus angulatus|CNC-HEM-0468
Hadronema militare|CNC-HEM-1332
Hadronema militare|CNC-HEM-1650
Ilnacora malina|CNC-HEM-0707
Ilnacora malina|CNC-HEM-0709
Ilnacora malina|CNC-HEM-0708
Mecomma gilvipes|CNC-HEM-0799
Mecomma gilvipes|CNC-HEM-0798
Mecomma gilvipes|CNC-HEM-0797
Orthotylus alni|CNC-HEM-0470
Orthotylus affinis|CNC-HEM-0585
Orthotylus affinis|CNC-HEM-0586
Orthotylus affinis|CNC-HEM-0587
Orthotylus alni|CNC-HEM-0472
Orthotylus pacificus|CNC-HEM-0791
Orthotylus pacificus|CNC-HEM-0792
Orthotylus katmai|CNC-HEM-0576
Orthotylus alni|CNC-HEM-0471
Orthotylus katmai|CNC-HEM-0577
Orthotylus pacificus|CNC-HEM-0793
Orthotylus katmai|CNC-HEM-0578
Orthotylus pacificus|CNC-HEM-0579
Aoplonema princeps|CNC-HEM-1651
Hadronema rubrum|CNC-HEM-1051
Hadronema rubrum|CNC-HEM-1050
Dryophilocoris flavoquadrimaculatus|CNC-HEM-0654
Dryophilocoris flavoquadrimaculatus|CNC-HEM-0655
Heterotoma planicornis|CNC-HEM-0771
Heterotoma planicornis|CNC-HEM-0772
Melymacra knighti|CNC-HEM-0691
Melymacra knighti|CNC-HEM-0692
Melymacra knighti|CNC-HEM-0693
Dichaetocoris piceicola|CNC-HEM-0704
Dichaetocoris piceicola|CNC-HEM-0703
Dichaetocoris piceicola|CNC-HEM-0705
Dichaetocoris piceicola|CNC-HEM-0706
Lopidea ampla|CNC-HEM-0603
Lopidea ampla|CNC-HEM-0604
Lopidea ampla|CNC-HEM-0602
Lopidea nigridia serica|CNC-HEM-0606
Lopidea nigridia serica|CNC-HEM-0607
Lopidea robiniae|CNC-HEM-0611
Lopidea robiniae|CNC-HEM-0612
Lopidea robiniae|CNC-HEM-0610
Lopidea instabilis|CNC-HEM-1649
Lopidea taurina|CNC-HEM-0608
Lopidea taurina|CNC-HEM-0609
Parthenicus wheeleri|CNC-HEM-0702
Parthenicus wheeleri|CNC-HEM-0701
Melanotrichus concolor|CNC-HEM-0795
Melanotrichus concolor|CNC-HEM-0796
Melanotrichus concolor|CNC-HEM-0794
Noctuocoris fumidus|CNC-HEM-0599
Noctuocoris fumidus|CNC-HEM-0600
Noctuocoris fumidus|CNC-HEM-0601
Scalponotatus maturus|CNC-HEM-0459
Scalponotatus maturus|CNC-HEM-0460
Scalponotatus maturus|CNC-HEM-0461
Slaterocoris ambrosiae|CNC-HEM-1122
Slaterocoris ambrosiae|CNC-HEM-1121
Slaterocoris rubrofemoratus|CNC-HEM-1125
Slaterocoris croceipes|CNC-HEM-1128
Slaterocoris croceipes|CNC-HEM-1127
Slaterocoris sparsus|CNC-HEM-1124
Slaterocoris breviatus|CNC-HEM-1131
Slaterocoris stygicus|CNC-HEM-1129
Slaterocoris stygicus|CNC-HEM-1130
Amblytylus nasutus|CNC-HEM-1503
Amblytylus nasutus|CNC-HEM-1623
Lepidargyrus ancorifer|CNC-HEM-0416
Lepidargyrus ancorifer|CNC-HEM-0414
Dichrooscytus repletus|HCNC376-09
Bolteria luteifrons|CNC-HEM-0349
Dichrooscytus elegans|CNC-HEM-1630
Dichrooscytus elegans|CNC-HEM-0367
Dichrooscytus elegans|CNC-HEM-0362
Dichrooscytus elegans|CNC-HEM-0363

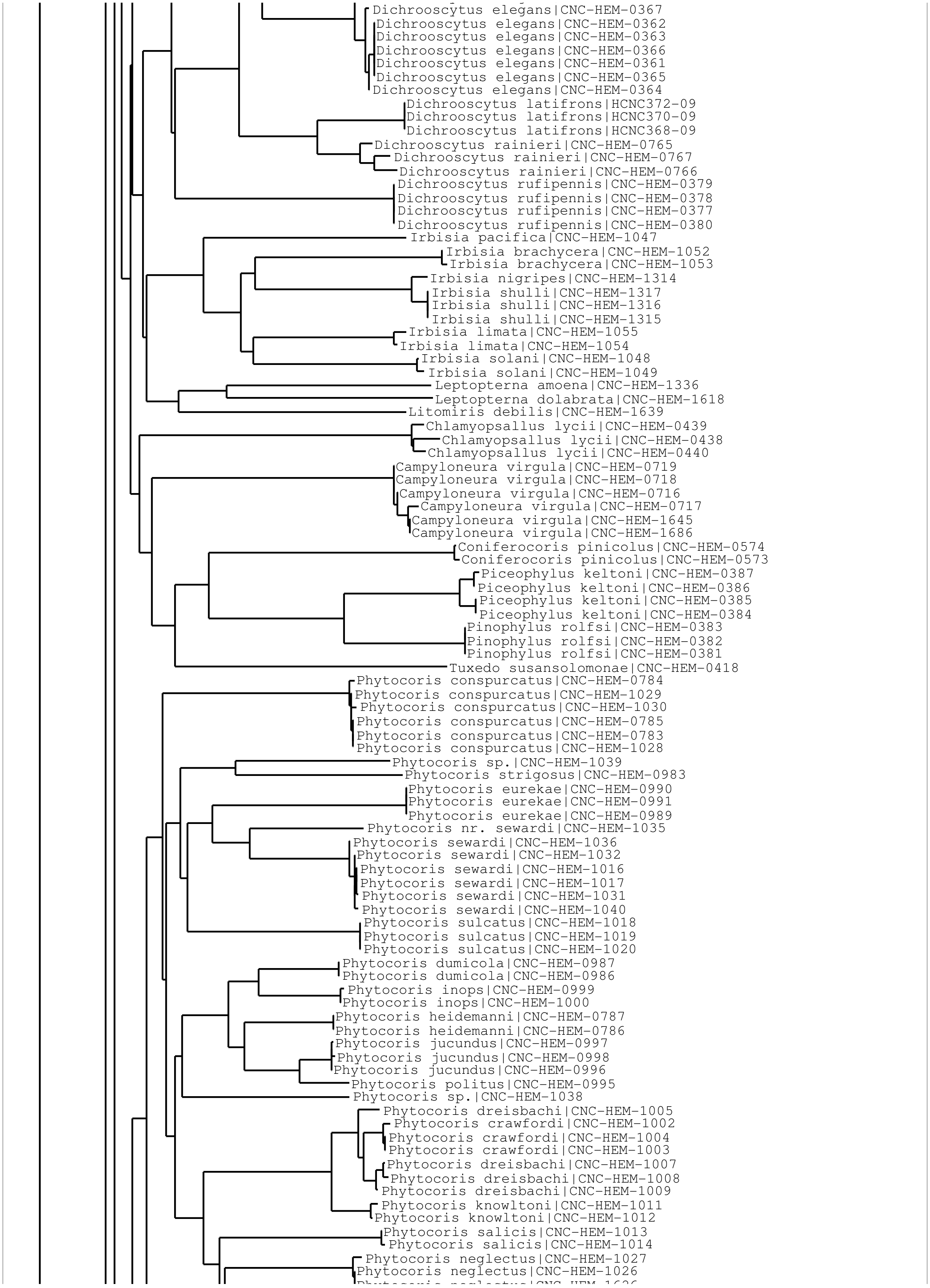
Dichrooscytus elegans|CNC-HEM-0367
Dichrooscytus elegans|CNC-HEM-0362
Dichrooscytus elegans|CNC-HEM-0363
Dichrooscytus elegans|CNC-HEM-0366
Dichrooscytus elegans|CNC-HEM-0361
Dichrooscytus elegans|CNC-HEM-0365
Dichrooscytus elegans|CNC-HEM-0364
Dichrooscytus latifrons|HCNC372-09
Dichrooscytus latifrons|HCNC370-09
Dichrooscytus latifrons|HCNC368-09
Dichrooscytus rainieri|CNC-HEM-0765
Dichrooscytus rainieri|CNC-HEM-0767
Dichrooscytus rainieri|CNC-HEM-0766
Dichrooscytus rufipennis|CNC-HEM-0379
Dichrooscytus rufipennis|CNC-HEM-0378
Dichrooscytus rufipennis|CNC-HEM-0377
Dichrooscytus rufipennis|CNC-HEM-0380
Irbisia pacifica|CNC-HEM-1047
Irbisia brachycera|CNC-HEM-1052
Irbisia brachycera|CNC-HEM-1053
Irbisia nigripes|CNC-HEM-1314
Irbisia shulli|CNC-HEM-1317
Irbisia shulli|CNC-HEM-1316
Irbisia shulli|CNC-HEM-1315
Irbisia limata|CNC-HEM-1055
Irbisia limata|CNC-HEM-1054
Irbisia solani|CNC-HEM-1048
Irbisia solani|CNC-HEM-1049
Leptopterna amoena|CNC-HEM-1336
Leptopterna dolabrata|CNC-HEM-1618
Litomiris debilis|CNC-HEM-1639
Chlamyopsallus lycii|CNC-HEM-0439
Chlamyopsallus lycii|CNC-HEM-0438
Chlamyopsallus lycii|CNC-HEM-0440
Campyloneura virgula|CNC-HEM-0719
Campyloneura virgula|CNC-HEM-0718
Campyloneura virgula|CNC-HEM-0716
Campyloneura virgula|CNC-HEM-0717
Campyloneura virgula|CNC-HEM-1645
Campyloneura virgula|CNC-HEM-1686
Coniferocoris pinicolus|CNC-HEM-0574
Coniferocoris pinicolus|CNC-HEM-0573
Piceophylus keltoni|CNC-HEM-0387
Piceophylus keltoni|CNC-HEM-0386
Piceophylus keltoni|CNC-HEM-0385
Piceophylus keltoni|CNC-HEM-0384
Pinophylus rolfsi|CNC-HEM-0383
Pinophylus rolfsi|CNC-HEM-0382
Pinophylus rolfsi|CNC-HEM-0381
Tuxedo susansolomonae|CNC-HEM-0418
Phytocoris conspurcatus|CNC-HEM-0784
Phytocoris conspurcatus|CNC-HEM-1029
Phytocoris conspurcatus|CNC-HEM-1030
Phytocoris conspurcatus|CNC-HEM-0785
Phytocoris conspurcatus|CNC-HEM-0783
Phytocoris conspurcatus|CNC-HEM-1028
Phytocoris sp.|CNC-HEM-1039
Phytocoris strigosus|CNC-HEM-0983
Phytocoris eurekae|CNC-HEM-0990
Phytocoris eurekae|CNC-HEM-0991
Phytocoris eurekae|CNC-HEM-0989
Phytocoris nr. sewardi|CNC-HEM-1035
Phytocoris sewardi|CNC-HEM-1036
Phytocoris sewardi|CNC-HEM-1032
Phytocoris sewardi|CNC-HEM-1016
Phytocoris sewardi|CNC-HEM-1017
Phytocoris sewardi|CNC-HEM-1031
Phytocoris sewardi|CNC-HEM-1040
Phytocoris sulcatus|CNC-HEM-1018
Phytocoris sulcatus|CNC-HEM-1019
Phytocoris sulcatus|CNC-HEM-1020
Phytocoris dumicola|CNC-HEM-0987
Phytocoris dumicola|CNC-HEM-0986
Phytocoris inops|CNC-HEM-0999
Phytocoris inops|CNC-HEM-1000
Phytocoris heidemanni|CNC-HEM-0787
Phytocoris heidemanni|CNC-HEM-0786
Phytocoris jucundus|CNC-HEM-0997
Phytocoris jucundus|CNC-HEM-0998
Phytocoris jucundus|CNC-HEM-0996
Phytocoris politus|CNC-HEM-0995
Phytocoris sp.|CNC-HEM-1038
Phytocoris dreisbachi|CNC-HEM-1005
Phytocoris crawfordi|CNC-HEM-1002
Phytocoris crawfordi|CNC-HEM-1004
Phytocoris crawfordi|CNC-HEM-1003
Phytocoris dreisbachi|CNC-HEM-1007
Phytocoris dreisbachi|CNC-HEM-1008
Phytocoris dreisbachi|CNC-HEM-1009
Phytocoris knowltoni|CNC-HEM-1011
Phytocoris knowltoni|CNC-HEM-1012
Phytocoris salicis|CNC-HEM-1013
Phytocoris salicis|CNC-HEM-1014
Phytocoris neglectus|CNC-HEM-1027
Phytocoris neglectus|CNC-HEM-1026

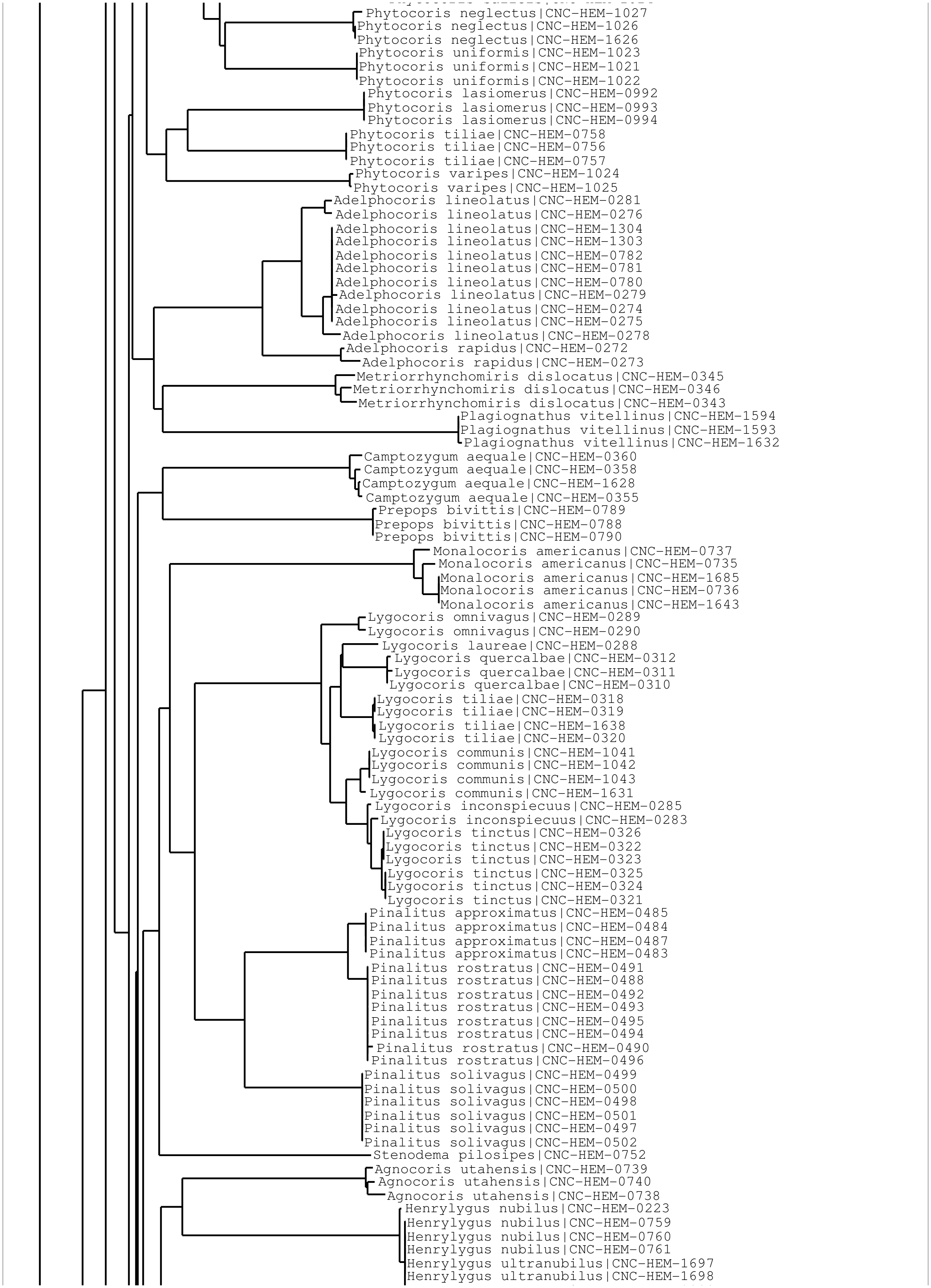

Phytocoris neglectus|CNC-HEM-1027
Phytocoris neglectus|CNC-HEM-1026
Phytocoris neglectus|CNC-HEM-1626
Phytocoris uniformis|CNC-HEM-1023
Phytocoris uniformis|CNC-HEM-1021
Phytocoris uniformis|CNC-HEM-1022
Phytocoris lasiomerus|CNC-HEM-0992
Phytocoris lasiomerus|CNC-HEM-0993
Phytocoris lasiomerus|CNC-HEM-0994
Phytocoris tiliae|CNC-HEM-0758
Phytocoris tiliae|CNC-HEM-0756
Phytocoris tiliae|CNC-HEM-0757
Phytocoris varipes|CNC-HEM-1024
Phytocoris varipes|CNC-HEM-1025
Adelphocoris lineolatus|CNC-HEM-0281
Adelphocoris lineolatus|CNC-HEM-0276
Adelphocoris lineolatus|CNC-HEM-1304
Adelphocoris lineolatus|CNC-HEM-1303
Adelphocoris lineolatus|CNC-HEM-0782
Adelphocoris lineolatus|CNC-HEM-0781
Adelphocoris lineolatus|CNC-HEM-0780
Adelphocoris lineolatus|CNC-HEM-0279
Adelphocoris lineolatus|CNC-HEM-0274
Adelphocoris lineolatus|CNC-HEM-0275
Adelphocoris lineolatus|CNC-HEM-0278
Adelphocoris rapidus|CNC-HEM-0272
Adelphocoris rapidus|CNC-HEM-0273
Metriorrhynchomiris dislocatus|CNC-HEM-0345
Metriorrhynchomiris dislocatus|CNC-HEM-0346
Metriorrhynchomiris dislocatus|CNC-HEM-0343
Plagiognathus vitellinus|CNC-HEM-1594
Plagiognathus vitellinus|CNC-HEM-1593
Plagiognathus vitellinus|CNC-HEM-1632
Camptozygum aequale|CNC-HEM-0360
Camptozygum aequale|CNC-HEM-0358
Camptozygum aequale|CNC-HEM-1628
Camptozygum aequale|CNC-HEM-0355
Prepops bivittis|CNC-HEM-0789
Prepops bivittis|CNC-HEM-0788
Prepops bivittis|CNC-HEM-0790
Monalocoris americanus|CNC-HEM-0737
Monalocoris americanus|CNC-HEM-0735
Monalocoris americanus|CNC-HEM-1685
Monalocoris americanus|CNC-HEM-0736
Monalocoris americanus|CNC-HEM-1643
Lygocoris omnivagus|CNC-HEM-0289
Lygocoris omnivagus|CNC-HEM-0290
Lygocoris laureae|CNC-HEM-0288
Lygocoris quercalbae|CNC-HEM-0312
Lygocoris quercalbae|CNC-HEM-0311
Lygocoris quercalbae|CNC-HEM-0310
Lygocoris tiliae|CNC-HEM-0318
Lygocoris tiliae|CNC-HEM-0319
Lygocoris tiliae|CNC-HEM-1638
Lygocoris tiliae|CNC-HEM-0320
Lygocoris communis|CNC-HEM-1041
Lygocoris communis|CNC-HEM-1042
Lygocoris communis|CNC-HEM-1043
Lygocoris communis|CNC-HEM-1631
Lygocoris inconspiecuus|CNC-HEM-0285
Lygocoris inconspiecuus|CNC-HEM-0283
Lygocoris tinctus|CNC-HEM-0326
Lygocoris tinctus|CNC-HEM-0322
Lygocoris tinctus|CNC-HEM-0323
Lygocoris tinctus|CNC-HEM-0325
Lygocoris tinctus|CNC-HEM-0324
Lygocoris tinctus|CNC-HEM-0321
Pinalitus approximatus|CNC-HEM-0485
Pinalitus approximatus|CNC-HEM-0484
Pinalitus approximatus|CNC-HEM-0487
Pinalitus approximatus|CNC-HEM-0483
Pinalitus rostratus|CNC-HEM-0491
Pinalitus rostratus|CNC-HEM-0488
Pinalitus rostratus|CNC-HEM-0492
Pinalitus rostratus|CNC-HEM-0493
Pinalitus rostratus|CNC-HEM-0495
Pinalitus rostratus|CNC-HEM-0494
Pinalitus rostratus|CNC-HEM-0490
Pinalitus rostratus|CNC-HEM-0496
Pinalitus solivagus|CNC-HEM-0499
Pinalitus solivagus|CNC-HEM-0500
Pinalitus solivagus|CNC-HEM-0498
Pinalitus solivagus|CNC-HEM-0501
Pinalitus solivagus|CNC-HEM-0497
Pinalitus solivagus|CNC-HEM-0502
Stenodema pilosipes|CNC-HEM-0752
Agnocoris utahensis|CNC-HEM-0739
Agnocoris utahensis|CNC-HEM-0740
Agnocoris utahensis|CNC-HEM-0738
Henrylygus nubilus|CNC-HEM-0223
Henrylygus nubilus|CNC-HEM-0759
Henrylygus nubilus|CNC-HEM-0760
Henrylygus nubilus|CNC-HEM-0761
Henrylygus ultranubilus|CNC-HEM-1697
Henrylygus ultranubilus|CNC-HEM-1698

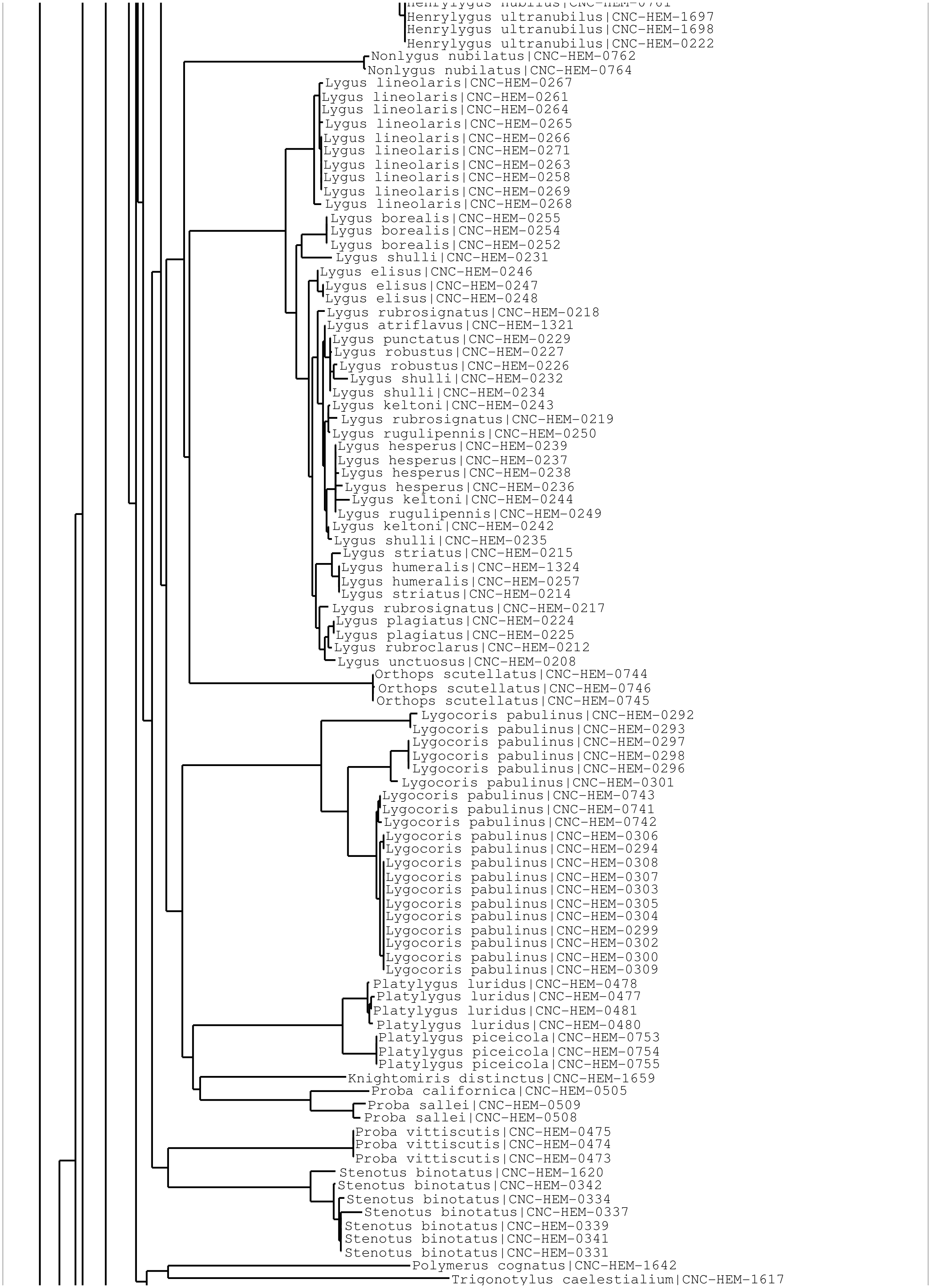
Henrylygus ultranubilus|CNC-HEM-1697
Henrylygus ultranubilus|CNC-HEM-1698
Henrylygus ultranubilus|CNC-HEM-0222
Nonlygus nubilatus|CNC-HEM-0762
Nonlygus nubilatus|CNC-HEM-0764
Lygus lineolaris|CNC-HEM-0267
Lygus lineolaris|CNC-HEM-0261
Lygus lineolaris|CNC-HEM-0264
Lygus lineolaris|CNC-HEM-0265
Lygus lineolaris|CNC-HEM-0266
Lygus lineolaris|CNC-HEM-0271
Lygus lineolaris|CNC-HEM-0263
Lygus lineolaris|CNC-HEM-0258
Lygus lineolaris|CNC-HEM-0269
Lygus lineolaris|CNC-HEM-0268
Lygus borealis|CNC-HEM-0255
Lygus borealis|CNC-HEM-0254
Lygus borealis|CNC-HEM-0252
Lygus shulli|CNC-HEM-0231
Lygus elisus|CNC-HEM-0246
Lygus elisus|CNC-HEM-0247
Lygus elisus|CNC-HEM-0248
Lygus rubrosignatus|CNC-HEM-0218
Lygus atriflavus|CNC-HEM-1321
Lygus punctatus|CNC-HEM-0229
Lygus robustus|CNC-HEM-0227
Lygus robustus|CNC-HEM-0226
Lygus shulli|CNC-HEM-0232
Lygus shulli|CNC-HEM-0234
Lygus keltoni|CNC-HEM-0243
Lygus rubrosignatus|CNC-HEM-0219
Lygus rugulipennis|CNC-HEM-0250
Lygus hesperus|CNC-HEM-0239
Lygus hesperus|CNC-HEM-0237
Lygus hesperus|CNC-HEM-0238
Lygus hesperus|CNC-HEM-0236
Lygus keltoni|CNC-HEM-0244
Lygus rugulipennis|CNC-HEM-0249
Lygus keltoni|CNC-HEM-0242
Lygus shulli|CNC-HEM-0235
Lygus striatus|CNC-HEM-0215
Lygus humeralis|CNC-HEM-1324
Lygus humeralis|CNC-HEM-0257
Lygus striatus|CNC-HEM-0214
Lygus rubrosignatus|CNC-HEM-0217
Lygus plagiatus|CNC-HEM-0224
Lygus plagiatus|CNC-HEM-0225
Lygus rubroclarus|CNC-HEM-0212
Lygus unctuosus|CNC-HEM-0208
Orthops scutellatus|CNC-HEM-0744
Orthops scutellatus|CNC-HEM-0746
Orthops scutellatus|CNC-HEM-0745
Lygocoris pabulinus|CNC-HEM-0292
Lygocoris pabulinus|CNC-HEM-0293
Lygocoris pabulinus|CNC-HEM-0297
Lygocoris pabulinus|CNC-HEM-0298
Lygocoris pabulinus|CNC-HEM-0296
Lygocoris pabulinus|CNC-HEM-0301
Lygocoris pabulinus|CNC-HEM-0743
Lygocoris pabulinus|CNC-HEM-0741
Lygocoris pabulinus|CNC-HEM-0742
Lygocoris pabulinus|CNC-HEM-0306
Lygocoris pabulinus|CNC-HEM-0294
Lygocoris pabulinus|CNC-HEM-0308
Lygocoris pabulinus|CNC-HEM-0307
Lygocoris pabulinus|CNC-HEM-0303
Lygocoris pabulinus|CNC-HEM-0305
Lygocoris pabulinus|CNC-HEM-0304
Lygocoris pabulinus|CNC-HEM-0299
Lygocoris pabulinus|CNC-HEM-0302
Lygocoris pabulinus|CNC-HEM-0300
Lygocoris pabulinus|CNC-HEM-0309
Platylygus luridus|CNC-HEM-0478
Platylygus luridus|CNC-HEM-0477
Platylygus luridus|CNC-HEM-0481
Platylygus luridus|CNC-HEM-0480
Platylygus piceicola|CNC-HEM-0753
Platylygus piceicola|CNC-HEM-0754
Platylygus piceicola|CNC-HEM-0755
Knightomiris distinctus|CNC-HEM-1659
Proba californica|CNC-HEM-0505
Proba sallei|CNC-HEM-0509
Proba sallei|CNC-HEM-0508
Proba vittiscutis|CNC-HEM-0475
Proba vittiscutis|CNC-HEM-0474
Proba vittiscutis|CNC-HEM-0473
Stenotus binotatus|CNC-HEM-1620
Stenotus binotatus|CNC-HEM-0342
Stenotus binotatus|CNC-HEM-0334
Stenotus binotatus|CNC-HEM-0337
Stenotus binotatus|CNC-HEM-0339
Stenotus binotatus|CNC-HEM-0341
Stenotus binotatus|CNC-HEM-0331
Polymerus cognatus|CNC-HEM-1642
Trigonotylus caelestialium|CNC-HEM-1617

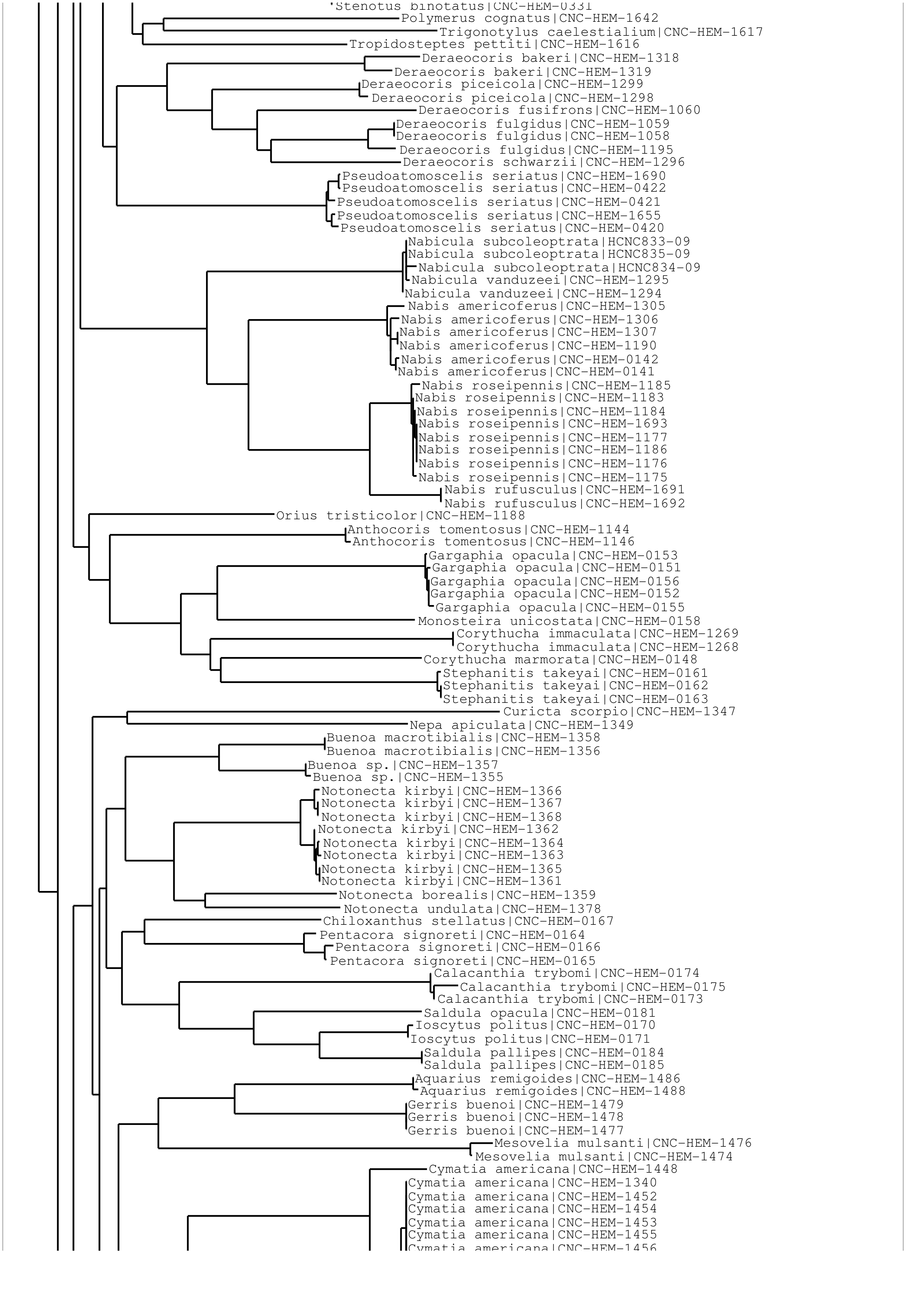
Stenotus binotatus|CNC-HEM-0331
Polymerus cognatus|CNC-HEM-1642
Trigonotylus caelestialium|CNC-HEM-1617
Tropidosteptes pettiti|CNC-HEM-1616
Deraeocoris bakeri|CNC-HEM-1318
Deraeocoris bakeri|CNC-HEM-1319
Deraeocoris piceicola|CNC-HEM-1299
Deraeocoris piceicola|CNC-HEM-1298
Deraeocoris fusifrons|CNC-HEM-1060
Deraeocoris fulgidus|CNC-HEM-1059
Deraeocoris fulgidus|CNC-HEM-1058
Deraeocoris fulgidus|CNC-HEM-1195
Deraeocoris schwarzii|CNC-HEM-1296
Pseudoatomoscelis seriatus|CNC-HEM-1690
Pseudoatomoscelis seriatus|CNC-HEM-0422
Pseudoatomoscelis seriatus|CNC-HEM-0421
Pseudoatomoscelis seriatus|CNC-HEM-1655
Pseudoatomoscelis seriatus|CNC-HEM-0420
Nabicula subcoleoptrata|HCNC833-09
Nabicula subcoleoptrata|HCNC835-09
Nabicula subcoleoptrata|HCNC834-09
Nabicula vanduzeei|CNC-HEM-1295
Nabicula vanduzeei|CNC-HEM-1294
Nabis americoferus|CNC-HEM-1305
Nabis americoferus|CNC-HEM-1306
Nabis americoferus|CNC-HEM-1307
Nabis americoferus|CNC-HEM-1190
Nabis americoferus|CNC-HEM-0142
Nabis americoferus|CNC-HEM-0141
Nabis roseipennis|CNC-HEM-1185
Nabis roseipennis|CNC-HEM-1183
Nabis roseipennis|CNC-HEM-1184
Nabis roseipennis|CNC-HEM-1693
Nabis roseipennis|CNC-HEM-1177
Nabis roseipennis|CNC-HEM-1186
Nabis roseipennis|CNC-HEM-1176
Nabis roseipennis|CNC-HEM-1175
Nabis rufusculus|CNC-HEM-1691
Nabis rufusculus|CNC-HEM-1692
Orius tristicolor|CNC-HEM-1188
Anthocoris tomentosus|CNC-HEM-1144
Anthocoris tomentosus|CNC-HEM-1146
Gargaphia opacula|CNC-HEM-0153
Gargaphia opacula|CNC-HEM-0151
Gargaphia opacula|CNC-HEM-0156
Gargaphia opacula|CNC-HEM-0152
Gargaphia opacula|CNC-HEM-0155
Monosteira unicostata|CNC-HEM-0158
Corythucha immaculata|CNC-HEM-1269
Corythucha immaculata|CNC-HEM-1268
Corythucha marmorata|CNC-HEM-0148
Stephanitis takeyai|CNC-HEM-0161
Stephanitis takeyai|CNC-HEM-0162
Stephanitis takeyai|CNC-HEM-0163
Curicta scorpio|CNC-HEM-1347
Nepa apiculata|CNC-HEM-1349
Buenoa macrotibialis|CNC-HEM-1358
Buenoa macrotibialis|CNC-HEM-1356
Buenoa sp.|CNC-HEM-1357
Buenoa sp.|CNC-HEM-1355
Notonecta kirbyi|CNC-HEM-1366
Notonecta kirbyi|CNC-HEM-1367
Notonecta kirbyi|CNC-HEM-1368
Notonecta kirbyi|CNC-HEM-1362
Notonecta kirbyi|CNC-HEM-1364
Notonecta kirbyi|CNC-HEM-1363
Notonecta kirbyi|CNC-HEM-1365
Notonecta kirbyi|CNC-HEM-1361
Notonecta borealis|CNC-HEM-1359
Notonecta undulata|CNC-HEM-1378
Chiloxanthus stellatus|CNC-HEM-0167
Pentacora signoreti|CNC-HEM-0164
Pentacora signoreti|CNC-HEM-0166
Pentacora signoreti|CNC-HEM-0165
Calacanthia trybomi|CNC-HEM-0174
Calacanthia trybomi|CNC-HEM-0175
Calacanthia trybomi|CNC-HEM-0173
Saldula opacula|CNC-HEM-0181
Ioscytus politus|CNC-HEM-0170
Ioscytus politus|CNC-HEM-0171
Saldula pallipes|CNC-HEM-0184
Saldula pallipes|CNC-HEM-0185
Aquarius remigoides|CNC-HEM-1486
Aquarius remigoides|CNC-HEM-1488
Gerris buenoi|CNC-HEM-1479
Gerris buenoi|CNC-HEM-1478
Gerris buenoi|CNC-HEM-1477
Mesovelia mulsanti|CNC-HEM-1476
Mesovelia mulsanti|CNC-HEM-1474
Cymatia americana|CNC-HEM-1448
Cymatia americana|CNC-HEM-1340
Cymatia americana|CNC-HEM-1452
Cymatia americana|CNC-HEM-1454
Cymatia americana|CNC-HEM-1453
Cymatia americana|CNC-HEM-1455
Cymatia americana|CNC-HEM-1456

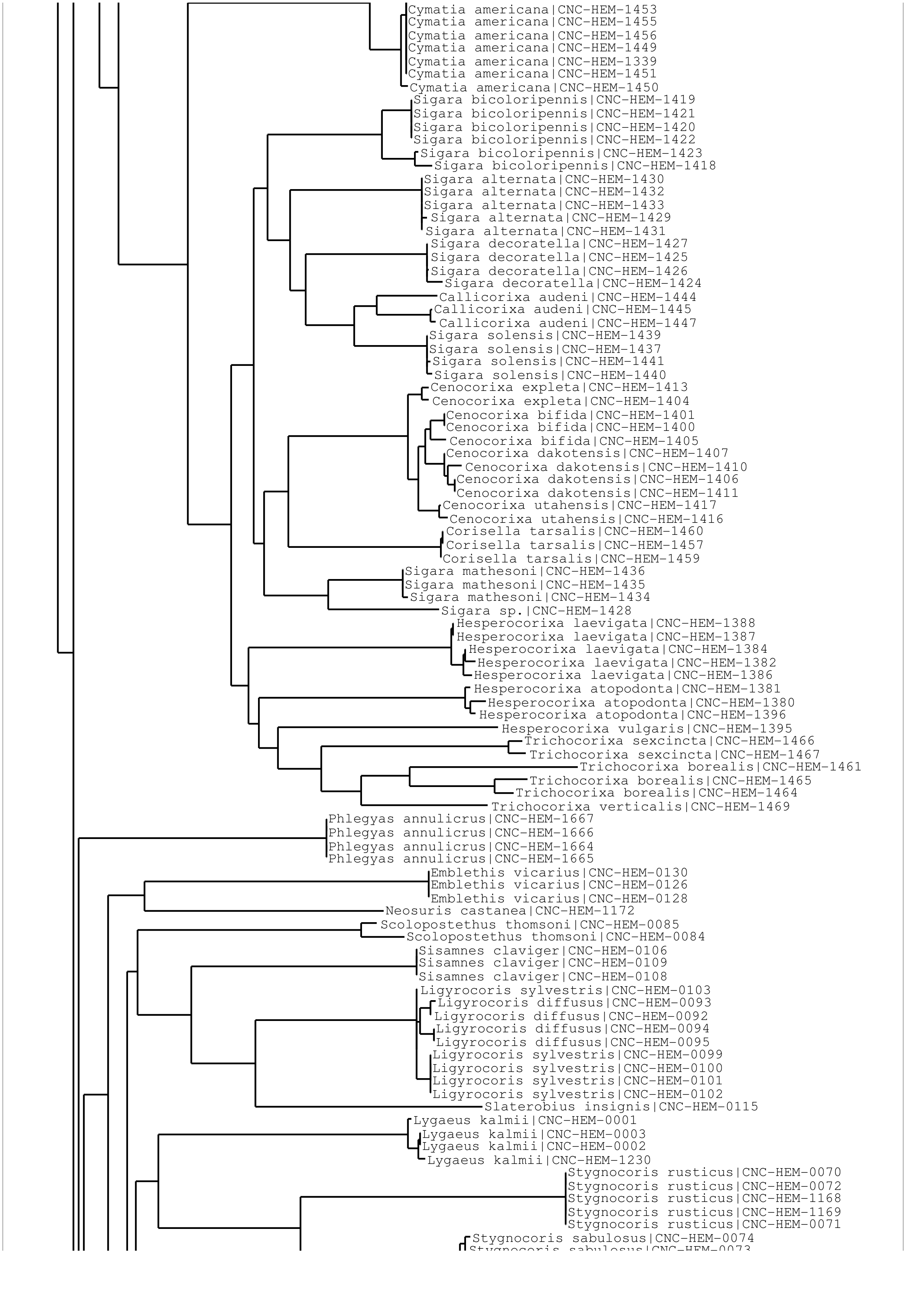
Cymatia americana|CNC-HEM-1453
Cymatia americana|CNC-HEM-1455
Cymatia americana|CNC-HEM-1456
Cymatia americana|CNC-HEM-1449
Cymatia americana|CNC-HEM-1339
Cymatia americana|CNC-HEM-1451
Cymatia americana|CNC-HEM-1450
Sigara bicoloripennis|CNC-HEM-1419
Sigara bicoloripennis|CNC-HEM-1421
Sigara bicoloripennis|CNC-HEM-1420
Sigara bicoloripennis|CNC-HEM-1422
Sigara bicoloripennis|CNC-HEM-1423
Sigara bicoloripennis|CNC-HEM-1418
Sigara alternata|CNC-HEM-1430
Sigara alternata|CNC-HEM-1432
Sigara alternata|CNC-HEM-1433
Sigara alternata|CNC-HEM-1429
Sigara alternata|CNC-HEM-1431
Sigara decoratella|CNC-HEM-1427
Sigara decoratella|CNC-HEM-1425
Sigara decoratella|CNC-HEM-1426
Sigara decoratella|CNC-HEM-1424
Callicorixa audeni|CNC-HEM-1444
Callicorixa audeni|CNC-HEM-1445
Callicorixa audeni|CNC-HEM-1447
Sigara solensis|CNC-HEM-1439
Sigara solensis|CNC-HEM-1437
Sigara solensis|CNC-HEM-1441
Sigara solensis|CNC-HEM-1440
Cenocorixa expleta|CNC-HEM-1413
Cenocorixa expleta|CNC-HEM-1404
Cenocorixa bifida|CNC-HEM-1401
Cenocorixa bifida|CNC-HEM-1400
Cenocorixa bifida|CNC-HEM-1405
Cenocorixa dakotensis|CNC-HEM-1407
Cenocorixa dakotensis|CNC-HEM-1410
Cenocorixa dakotensis|CNC-HEM-1406
Cenocorixa dakotensis|CNC-HEM-1411
Cenocorixa utahensis|CNC-HEM-1417
Cenocorixa utahensis|CNC-HEM-1416
Corisella tarsalis|CNC-HEM-1460
Corisella tarsalis|CNC-HEM-1457
Corisella tarsalis|CNC-HEM-1459
Sigara mathesoni|CNC-HEM-1436
Sigara mathesoni|CNC-HEM-1435
Sigara mathesoni|CNC-HEM-1434
Sigara sp.|CNC-HEM-1428
Hesperocorixa laevigata|CNC-HEM-1388
Hesperocorixa laevigata|CNC-HEM-1387
Hesperocorixa laevigata|CNC-HEM-1384
Hesperocorixa laevigata|CNC-HEM-1382
Hesperocorixa laevigata|CNC-HEM-1386
Hesperocorixa atopodonta|CNC-HEM-1381
Hesperocorixa atopodonta|CNC-HEM-1380
Hesperocorixa atopodonta|CNC-HEM-1396
Hesperocorixa vulgaris|CNC-HEM-1395
Trichocorixa sexcincta|CNC-HEM-1466
Trichocorixa sexcincta|CNC-HEM-1467
Trichocorixa borealis|CNC-HEM-1461
Trichocorixa borealis|CNC-HEM-1465
Trichocorixa borealis|CNC-HEM-1464
Trichocorixa verticalis|CNC-HEM-1469
Phlegyas annulicrus|CNC-HEM-1667
Phlegyas annulicrus|CNC-HEM-1666
Phlegyas annulicrus|CNC-HEM-1664
Phlegyas annulicrus|CNC-HEM-1665
Emblethis vicarius|CNC-HEM-0130
Emblethis vicarius|CNC-HEM-0126
Emblethis vicarius|CNC-HEM-0128
Neosuris castanea|CNC-HEM-1172
Scolopostethus thomsoni|CNC-HEM-0085
Scolopostethus thomsoni|CNC-HEM-0084
Sisamnes claviger|CNC-HEM-0106
Sisamnes claviger|CNC-HEM-0109
Sisamnes claviger|CNC-HEM-0108
Ligyrocoris sylvestris|CNC-HEM-0103
Ligyrocoris diffusus|CNC-HEM-0093
Ligyrocoris diffusus|CNC-HEM-0092
Ligyrocoris diffusus|CNC-HEM-0094
Ligyrocoris diffusus|CNC-HEM-0095
Ligyrocoris sylvestris|CNC-HEM-0099
Ligyrocoris sylvestris|CNC-HEM-0100
Ligyrocoris sylvestris|CNC-HEM-0101
Ligyrocoris sylvestris|CNC-HEM-0102
Slaterobius insignis|CNC-HEM-0115
Lygaeus kalmii|CNC-HEM-0001
Lygaeus kalmii|CNC-HEM-0003
Lygaeus kalmii|CNC-HEM-0002
Lygaeus kalmii|CNC-HEM-1230
Stygnocoris rusticus|CNC-HEM-0070
Stygnocoris rusticus|CNC-HEM-0072
Stygnocoris rusticus|CNC-HEM-1168
Stygnocoris rusticus|CNC-HEM-1169
Stygnocoris rusticus|CNC-HEM-0071
Stygnocoris sabulosus|CNC-HEM-0074

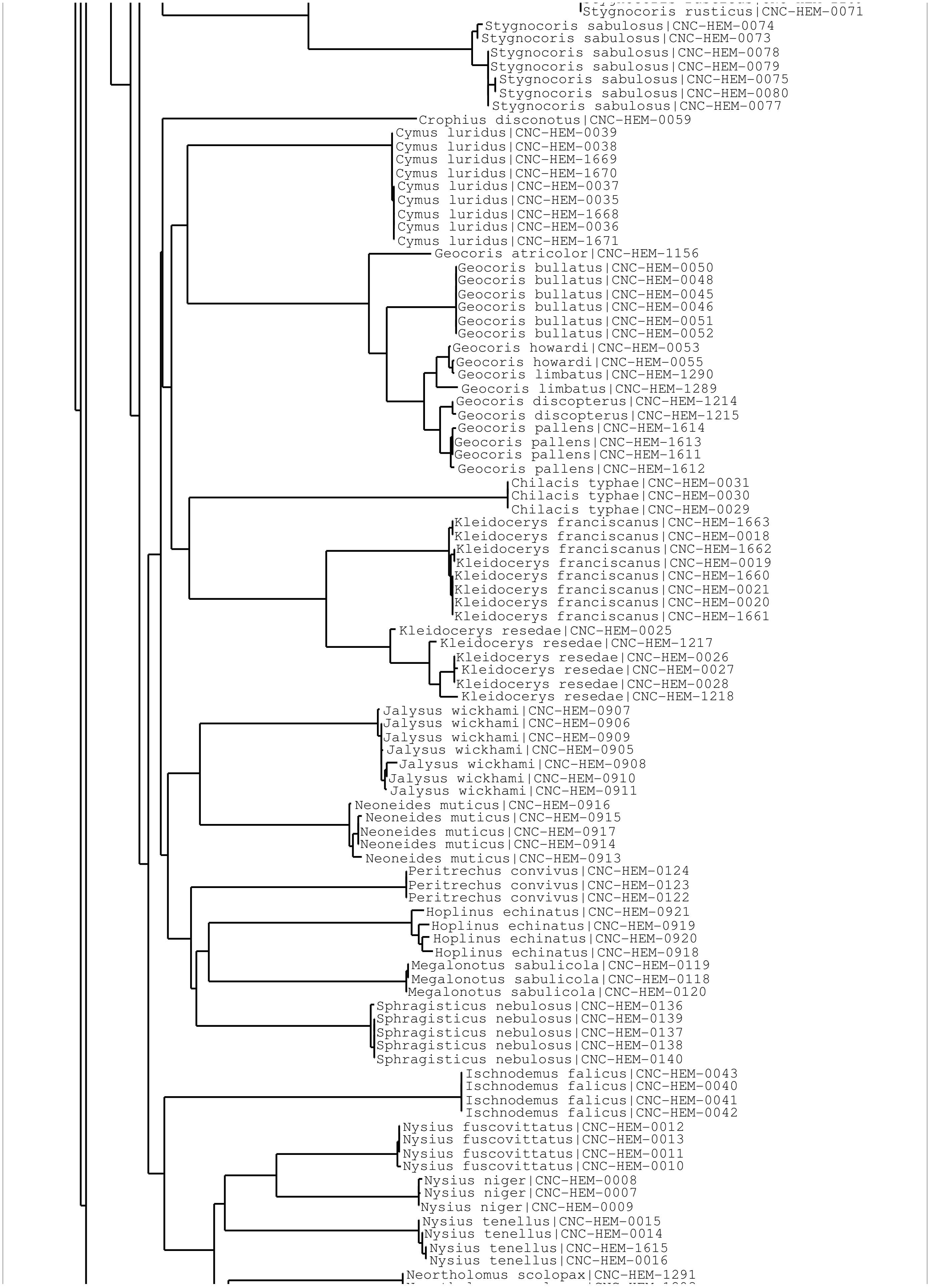
Stygnocoris rusticus|CNC-HEM-0071
Stygnocoris sabulosus|CNC-HEM-0074
Stygnocoris sabulosus|CNC-HEM-0073
Stygnocoris sabulosus|CNC-HEM-0078
Stygnocoris sabulosus|CNC-HEM-0079
Stygnocoris sabulosus|CNC-HEM-0075
Stygnocoris sabulosus|CNC-HEM-0080
Stygnocoris sabulosus|CNC-HEM-0077
Crophius disconotus|CNC-HEM-0059
Cymus luridus|CNC-HEM-0039
Cymus luridus|CNC-HEM-0038
Cymus luridus|CNC-HEM-1669
Cymus luridus|CNC-HEM-1670
Cymus luridus|CNC-HEM-0037
Cymus luridus|CNC-HEM-0035
Cymus luridus|CNC-HEM-1668
Cymus luridus|CNC-HEM-0036
Cymus luridus|CNC-HEM-1671
Geocoris atricolor|CNC-HEM-1156
Geocoris bullatus|CNC-HEM-0050
Geocoris bullatus|CNC-HEM-0048
Geocoris bullatus|CNC-HEM-0045
Geocoris bullatus|CNC-HEM-0046
Geocoris bullatus|CNC-HEM-0051
Geocoris bullatus|CNC-HEM-0052
Geocoris howardi|CNC-HEM-0053
Geocoris howardi|CNC-HEM-0055
Geocoris limbatus|CNC-HEM-1290
Geocoris limbatus|CNC-HEM-1289
Geocoris discopterus|CNC-HEM-1214
Geocoris discopterus|CNC-HEM-1215
Geocoris pallens|CNC-HEM-1614
Geocoris pallens|CNC-HEM-1613
Geocoris pallens|CNC-HEM-1611
Geocoris pallens|CNC-HEM-1612
Chilacis typhae|CNC-HEM-0031
Chilacis typhae|CNC-HEM-0030
Chilacis typhae|CNC-HEM-0029
Kleidocerys franciscanus|CNC-HEM-1663
Kleidocerys franciscanus|CNC-HEM-0018
Kleidocerys franciscanus|CNC-HEM-1662
Kleidocerys franciscanus|CNC-HEM-0019
Kleidocerys franciscanus|CNC-HEM-1660
Kleidocerys franciscanus|CNC-HEM-0021
Kleidocerys franciscanus|CNC-HEM-0020
Kleidocerys franciscanus|CNC-HEM-1661
Kleidocerys resedae|CNC-HEM-0025
Kleidocerys resedae|CNC-HEM-1217
Kleidocerys resedae|CNC-HEM-0026
Kleidocerys resedae|CNC-HEM-0027
Kleidocerys resedae|CNC-HEM-0028
Kleidocerys resedae|CNC-HEM-1218
Jalysus wickhami|CNC-HEM-0907
Jalysus wickhami|CNC-HEM-0906
Jalysus wickhami|CNC-HEM-0909
Jalysus wickhami|CNC-HEM-0905
Jalysus wickhami|CNC-HEM-0908
Jalysus wickhami|CNC-HEM-0910
Jalysus wickhami|CNC-HEM-0911
Neoneides muticus|CNC-HEM-0916
Neoneides muticus|CNC-HEM-0915
Neoneides muticus|CNC-HEM-0917
Neoneides muticus|CNC-HEM-0914
Neoneides muticus|CNC-HEM-0913
Peritrechus convivus|CNC-HEM-0124
Peritrechus convivus|CNC-HEM-0123
Peritrechus convivus|CNC-HEM-0122
Hoplinus echinatus|CNC-HEM-0921
Hoplinus echinatus|CNC-HEM-0919
Hoplinus echinatus|CNC-HEM-0920
Hoplinus echinatus|CNC-HEM-0918
Megalonotus sabulicola|CNC-HEM-0119
Megalonotus sabulicola|CNC-HEM-0118
Megalonotus sabulicola|CNC-HEM-0120
Sphragisticus nebulosus|CNC-HEM-0136
Sphragisticus nebulosus|CNC-HEM-0139
Sphragisticus nebulosus|CNC-HEM-0137
Sphragisticus nebulosus|CNC-HEM-0138
Sphragisticus nebulosus|CNC-HEM-0140
Ischnodemus falicus|CNC-HEM-0043
Ischnodemus falicus|CNC-HEM-0040
Ischnodemus falicus|CNC-HEM-0041
Ischnodemus falicus|CNC-HEM-0042
Nysius fuscovittatus|CNC-HEM-0012
Nysius fuscovittatus|CNC-HEM-0013
Nysius fuscovittatus|CNC-HEM-0011
Nysius fuscovittatus|CNC-HEM-0010
Nysius niger|CNC-HEM-0008
Nysius niger|CNC-HEM-0007
Nysius niger|CNC-HEM-0009
Nysius tenellus|CNC-HEM-0015
Nysius tenellus|CNC-HEM-0014
Nysius tenellus|CNC-HEM-1615
Nysius tenellus|CNC-HEM-0016
Neortholomus scolopax|CNC-HEM-1291

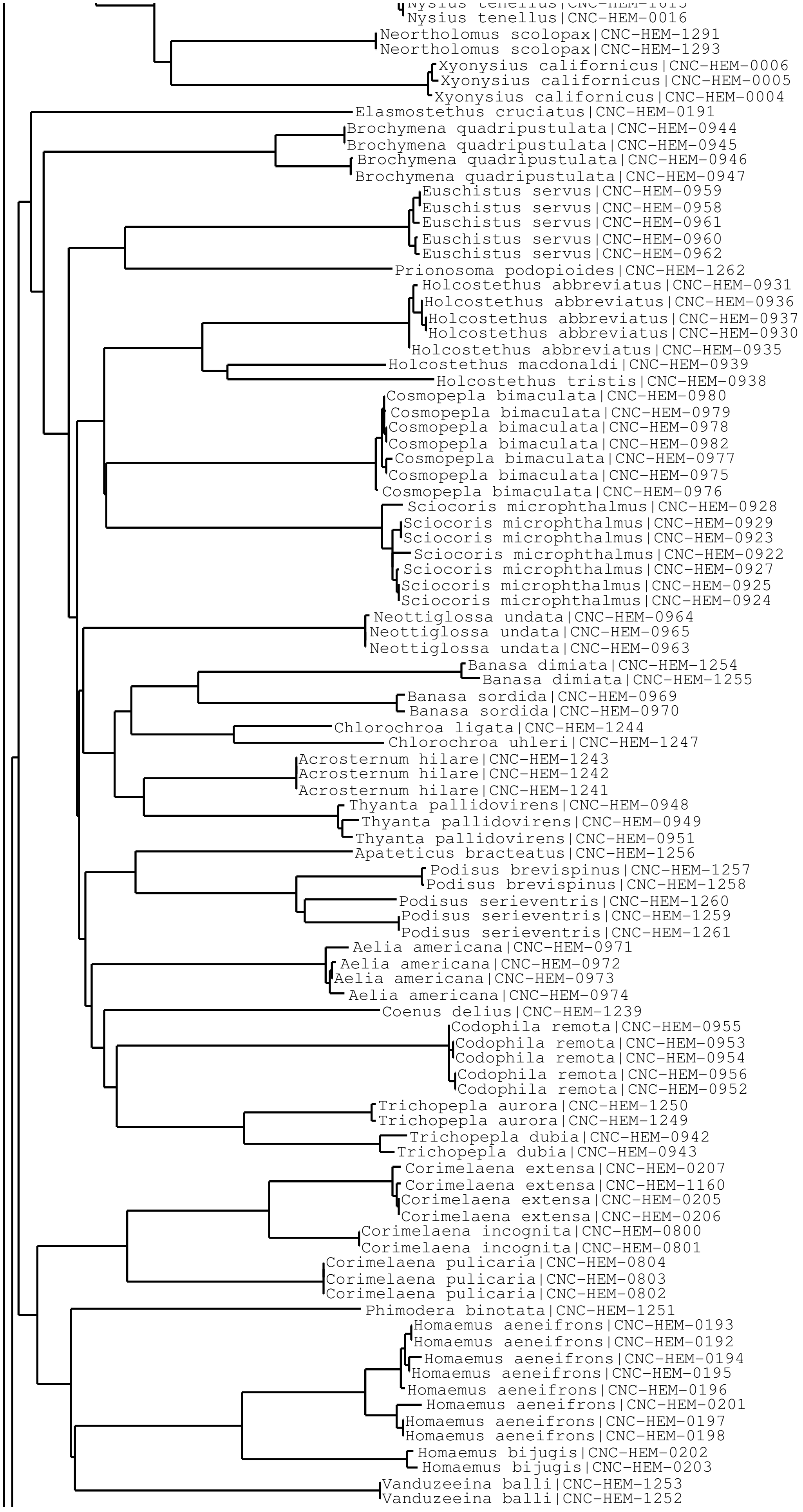
Nysius tenellus|CNC-HEM-0016
Neortholomus scolopax|CNC-HEM-1291
Neortholomus scolopax|CNC-HEM-1293
Xyonysius californicus|CNC-HEM-0006
Xyonysius californicus|CNC-HEM-0005
Xyonysius californicus|CNC-HEM-0004
Elasmostethus cruciatus|CNC-HEM-0191
Brochymena quadripustulata|CNC-HEM-0944
Brochymena quadripustulata|CNC-HEM-0945
Brochymena quadripustulata|CNC-HEM-0946
Brochymena quadripustulata|CNC-HEM-0947
Euschistus servus|CNC-HEM-0959
Euschistus servus|CNC-HEM-0958
Euschistus servus|CNC-HEM-0961
Euschistus servus|CNC-HEM-0960
Euschistus servus|CNC-HEM-0962
Prionosoma podopioides|CNC-HEM-1262
Holcostethus abbreviatus|CNC-HEM-0931
Holcostethus abbreviatus|CNC-HEM-0936
Holcostethus abbreviatus|CNC-HEM-0937
Holcostethus abbreviatus|CNC-HEM-0930
Holcostethus abbreviatus|CNC-HEM-0935
Holcostethus macdonaldi|CNC-HEM-0939
Holcostethus tristis|CNC-HEM-0938
Cosmopepla bimaculata|CNC-HEM-0980
Cosmopepla bimaculata|CNC-HEM-0979
Cosmopepla bimaculata|CNC-HEM-0978
Cosmopepla bimaculata|CNC-HEM-0982
Cosmopepla bimaculata|CNC-HEM-0977
Cosmopepla bimaculata|CNC-HEM-0975
Cosmopepla bimaculata|CNC-HEM-0976
Sciocoris microphthalmus|CNC-HEM-0928
Sciocoris microphthalmus|CNC-HEM-0929
Sciocoris microphthalmus|CNC-HEM-0923
Sciocoris microphthalmus|CNC-HEM-0922
Sciocoris microphthalmus|CNC-HEM-0927
Sciocoris microphthalmus|CNC-HEM-0925
Sciocoris microphthalmus|CNC-HEM-0924
Neottiglossa undata|CNC-HEM-0964
Neottiglossa undata|CNC-HEM-0965
Neottiglossa undata|CNC-HEM-0963
Banasa dimiata|CNC-HEM-1254
Banasa dimiata|CNC-HEM-1255
Banasa sordida|CNC-HEM-0969
Banasa sordida|CNC-HEM-0970
Chlorochroa ligata|CNC-HEM-1244
Chlorochroa uhleri|CNC-HEM-1247
Acrosternum hilare|CNC-HEM-1243
Acrosternum hilare|CNC-HEM-1242
Acrosternum hilare|CNC-HEM-1241
Thyanta pallidovirens|CNC-HEM-0948
Thyanta pallidovirens|CNC-HEM-0949
Thyanta pallidovirens|CNC-HEM-0951
Apateticus bracteatus|CNC-HEM-1256
Podisus brevispinus|CNC-HEM-1257
Podisus brevispinus|CNC-HEM-1258
Podisus serieventris|CNC-HEM-1260
Podisus serieventris|CNC-HEM-1259
Podisus serieventris|CNC-HEM-1261
Aelia americana|CNC-HEM-0971
Aelia americana|CNC-HEM-0972
Aelia americana|CNC-HEM-0973
Aelia americana|CNC-HEM-0974
Coenus delius|CNC-HEM-1239
Codophila remota|CNC-HEM-0955
Codophila remota|CNC-HEM-0953
Codophila remota|CNC-HEM-0954
Codophila remota|CNC-HEM-0956
Codophila remota|CNC-HEM-0952
Trichopepla aurora|CNC-HEM-1250
Trichopepla aurora|CNC-HEM-1249
Trichopepla dubia|CNC-HEM-0942
Trichopepla dubia|CNC-HEM-0943
Corimelaena extensa|CNC-HEM-0207
Corimelaena extensa|CNC-HEM-1160
Corimelaena extensa|CNC-HEM-0205
Corimelaena extensa|CNC-HEM-0206
Corimelaena incognita|CNC-HEM-0800
Corimelaena incognita|CNC-HEM-0801
Corimelaena pulicaria|CNC-HEM-0804
Corimelaena pulicaria|CNC-HEM-0803
Corimelaena pulicaria|CNC-HEM-0802
Phimodera binotata|CNC-HEM-1251
Homaemus aeneifrons|CNC-HEM-0193
Homaemus aeneifrons|CNC-HEM-0192
Homaemus aeneifrons|CNC-HEM-0194
Homaemus aeneifrons|CNC-HEM-0195
Homaemus aeneifrons|CNC-HEM-0196
Homaemus aeneifrons|CNC-HEM-0201
Homaemus aeneifrons|CNC-HEM-0197
Homaemus aeneifrons|CNC-HEM-0198
Homaemus bijugis|CNC-HEM-0202
Homaemus bijugis|CNC-HEM-0203
Vanduzeeina balli|CNC-HEM-1253
Vanduzeeina balli|CNC-HEM-1252

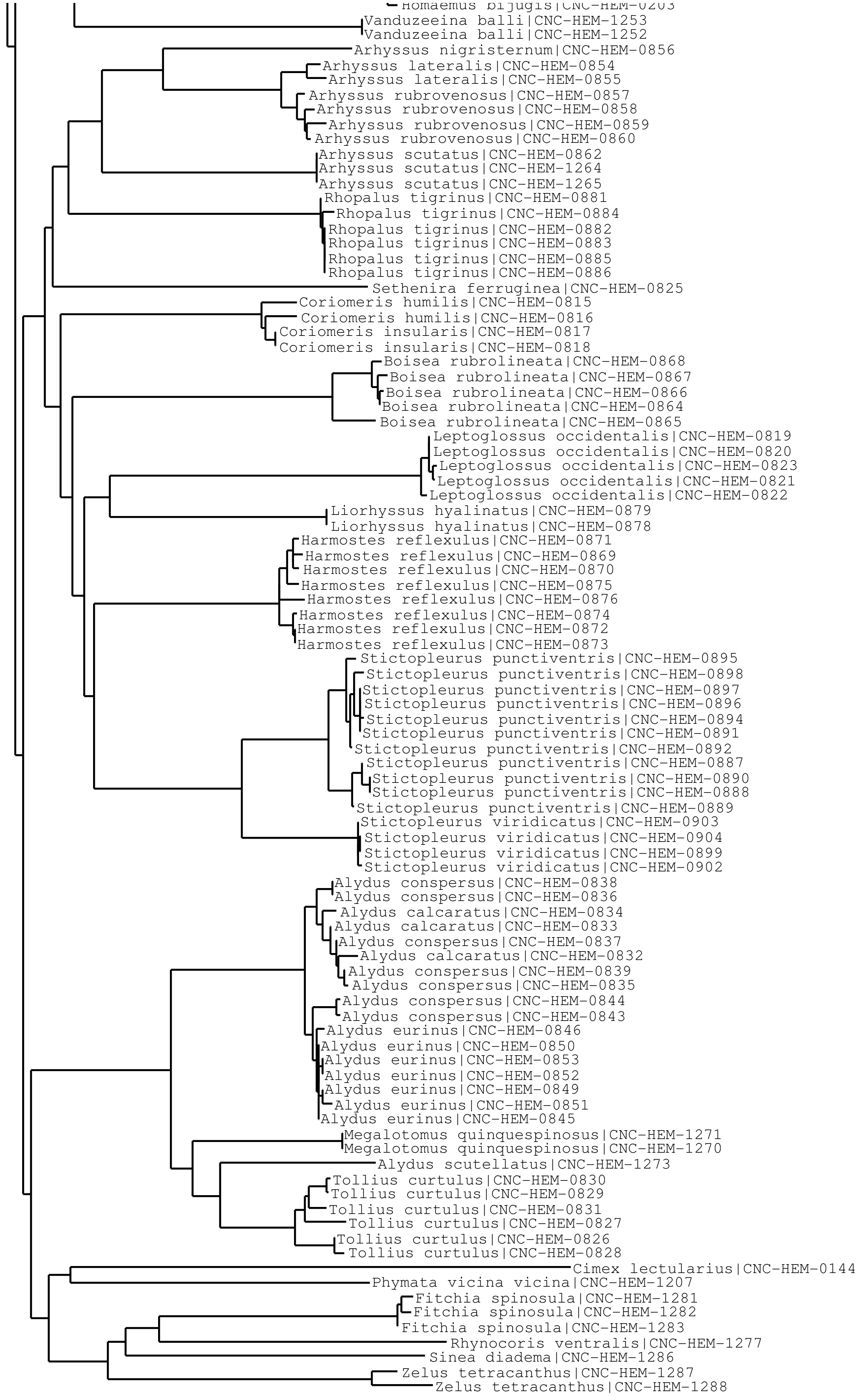
Vanduzeeina balli|CNC-HEM-1253
Vanduzeeina balli|CNC-HEM-1252
Arhyssus nigristernum|CNC-HEM-0856
Arhyssus lateralis|CNC-HEM-0854
Arhyssus lateralis|CNC-HEM-0855
Arhyssus rubrovenosus|CNC-HEM-0857
Arhyssus rubrovenosus|CNC-HEM-0858
Arhyssus rubrovenosus|CNC-HEM-0859
Arhyssus rubrovenosus|CNC-HEM-0860
Arhyssus scutatus|CNC-HEM-0862
Arhyssus scutatus|CNC-HEM-1264
Arhyssus scutatus|CNC-HEM-1265
Rhopalus tigrinus|CNC-HEM-0881
Rhopalus tigrinus|CNC-HEM-0884
Rhopalus tigrinus|CNC-HEM-0882
Rhopalus tigrinus|CNC-HEM-0883
Rhopalus tigrinus|CNC-HEM-0885
Rhopalus tigrinus|CNC-HEM-0886
Sethenira ferruginea|CNC-HEM-0825
Coriomeris humilis|CNC-HEM-0815
Coriomeris humilis|CNC-HEM-0816
Coriomeris insularis|CNC-HEM-0817
Coriomeris insularis|CNC-HEM-0818
Boisea rubrolineata|CNC-HEM-0868
Boisea rubrolineata|CNC-HEM-0867
Boisea rubrolineata|CNC-HEM-0866
Boisea rubrolineata|CNC-HEM-0864
Boisea rubrolineata|CNC-HEM-0865
Leptoglossus occidentalis|CNC-HEM-0819
Leptoglossus occidentalis|CNC-HEM-0820
Leptoglossus occidentalis|CNC-HEM-0823
Leptoglossus occidentalis|CNC-HEM-0821
Leptoglossus occidentalis|CNC-HEM-0822
Liorhyssus hyalinatus|CNC-HEM-0879
Liorhyssus hyalinatus|CNC-HEM-0878
Harmostes reflexulus|CNC-HEM-0871
Harmostes reflexulus|CNC-HEM-0869
Harmostes reflexulus|CNC-HEM-0870
Harmostes reflexulus|CNC-HEM-0875
Harmostes reflexulus|CNC-HEM-0876
Harmostes reflexulus|CNC-HEM-0874
Harmostes reflexulus|CNC-HEM-0872
Harmostes reflexulus|CNC-HEM-0873
Stictopleurus punctiventris|CNC-HEM-0895
Stictopleurus punctiventris|CNC-HEM-0898
Stictopleurus punctiventris|CNC-HEM-0897
Stictopleurus punctiventris|CNC-HEM-0896
Stictopleurus punctiventris|CNC-HEM-0894
Stictopleurus punctiventris|CNC-HEM-0891
Stictopleurus punctiventris|CNC-HEM-0892
Stictopleurus punctiventris|CNC-HEM-0887
Stictopleurus punctiventris|CNC-HEM-0890
Stictopleurus punctiventris|CNC-HEM-0888
Stictopleurus punctiventris|CNC-HEM-0889
Stictopleurus viridicatus|CNC-HEM-0903
Stictopleurus viridicatus|CNC-HEM-0904
Stictopleurus viridicatus|CNC-HEM-0899
Stictopleurus viridicatus|CNC-HEM-0902
Alydus conspersus|CNC-HEM-0838
Alydus conspersus|CNC-HEM-0836
Alydus calcaratus|CNC-HEM-0834
Alydus calcaratus|CNC-HEM-0833
Alydus conspersus|CNC-HEM-0837
Alydus calcaratus|CNC-HEM-0832
Alydus conspersus|CNC-HEM-0839
Alydus conspersus|CNC-HEM-0835
Alydus conspersus|CNC-HEM-0844
Alydus conspersus|CNC-HEM-0843
Alydus eurinus|CNC-HEM-0846
Alydus eurinus|CNC-HEM-0850
Alydus eurinus|CNC-HEM-0853
Alydus eurinus|CNC-HEM-0852
Alydus eurinus|CNC-HEM-0849
Alydus eurinus|CNC-HEM-0851
Alydus eurinus|CNC-HEM-0845
Megalotomus quinquespinosus|CNC-HEM-1271
Megalotomus quinquespinosus|CNC-HEM-1270
Alydus scutellatus|CNC-HEM-1273
Tollius curtulus|CNC-HEM-0830
Tollius curtulus|CNC-HEM-0829
Tollius curtulus|CNC-HEM-0831
Tollius curtulus|CNC-HEM-0827
Tollius curtulus|CNC-HEM-0826
Tollius curtulus|CNC-HEM-0828
Cimex lectularius|CNC-HEM-0144
Phymata vicina vicina|CNC-HEM-1207
Fitchia spinosula|CNC-HEM-1281
Fitchia spinosula|CNC-HEM-1282
Fitchia spinosula|CNC-HEM-1283
Rhynocoris ventralis|CNC-HEM-1277
Sinea diadema|CNC-HEM-1286
Zelus tetracanthus|CNC-HEM-1287
Zelus tetracanthus|CNC-HEM-1288
